# Diversity and Classification of the Actinopeptins: A New Family of Lanthipeptides Within the Genomes from the Phylum Actinomycetota

**DOI:** 10.3390/antibiotics15080755

**Published:** 2026-08-05

**Authors:** Carlos García-Ausencio, Beatriz Ruiz-Villafán, Adelfo Escalante-Lozada, Martha Lydia Macías-Rubalcava, Romina Rodríguez-Sanoja, Sergio Sánchez

**Affiliations:** 1 Departamento de Biología Molecular y Biotecnología, Instituto de Investigaciones Biomédicas, Universidad Nacional Autónoma de México (UNAM), Mexico City 04510, Mexico; beatrizruiz@biomedicas.unam.mx (B.R.-V.); romina@iibiomedicas.unam.mx (R.R.-S.); 2 Departamento de Ingeniería Celular y Biocatálisis, Instituto de Biotecnología, Universidad Nacional Autónoma de México (UNAM), Cuernavaca 62210, Mexico; adelfo.escalante@ibt.unam.mx; 3 Departamento de Productos Naturales, Instituto de Química, Universidad Nacional Autónoma de México (UNAM), Mexico City 04510, Mexico; mmacias@iquimica.unam.mx

**Keywords:** RiPPs, lanthipeptides, genome mining, DUF6229, actinopeptins

## Abstract

Background/Objectives: Lanthipeptides are post-translationally modified compounds and have broad industrial potential. Six distinct classes of lanthipeptides are defined by the biosynthetic enzymes that install lanthionine and β-methyl-lanthionine. Notably, class II lanthipeptides are modified by the LanM enzyme and are encoded by specific biosynthetic clusters found in both Gram-positive and Gram-negative bacteria. Among bacterial phyla, the Actinomycetota is particularly notable for encoding a rich diversity of lanthipeptides. In this study, we investigated the diversity of a new class II lanthipeptide group, the actinopeptin family, within the phylum Actinomycetota. Methods: Using genome-mining tools, we analyzed 85 producer genomes encoding actinopeptins. Results: When comparing clusters, we found that high overall similarity is restricted to only a few sequences, whereas most clusters display marked differences, which highlights the broad diversity of this lanthipeptide family. Our comparative analysis revealed that while the leader peptide is conserved across this group, core peptides are highly diverse. Conclusions: This finding allowed us to categorize the entire family into distinct groups. We also identified clusters encoding multiple peptides modified by LanM, suggesting that it may exhibit substrate promiscuity. This study highlights the actinopeptin family as a promising source of new compounds.

## 1. Introduction

Lanthipeptides are ribosomally synthesized and post-translationally modified peptides (RiPPs) present in various bacterial taxa [1,2,3,4]. They are characterized by the presence of lanthionine (Lan) and β-methyl-lanthionine (MeLan) rings in the peptide LanA. This peptide consists of a leader and a core peptide. The rings result from dehydration and cyclization reactions that involve serine and threonine amino acids in the core peptide, while the leader is the region that binds to specialized enzymes that catalyze these modifications [5,6]. The LanM enzyme specifically catalyzes ring formation in class II lanthipeptides. It consists of two key regions: a N-terminal dehydration domain, annotated as “domain of unknown function 4135” (DUF4135), which is unique to these ATP- and magnesium-dependent enzymes, and a C-terminal region that facilitates the cyclization reaction through zinc chelation [6,7,8]. In certain cases, after the rings are formed, additional tailoring enzymes further modify the peptide by adding functional groups such as hydroxyls or lysinoalanine linkages [9,10,11]. The resulting modified peptide, known as the mature peptide, can display antiviral and anticancer properties [4,12,13], but the most outstanding feature is its antibacterial activity.

Class II lanthipeptides, like mersacidin produced by *Bacillus* sp. HIL Y-85,54728, are characterized by binding to the cell wall precursor lipid II. Unlike clinical antibiotics, this interaction occurs through interaction with the pyrophosphate bridge of lipid II, which decreases the probability of antibiotic bacterial resistance [14,15,16]. In this sense, many lantibiotics (antibiotics containing lanthionine) are effective against multidrug-resistant bacteria (MDR) such as methicillin-resistant *Staphylococcus aureus* (MRSA) and vancomycin-resistant enterococci (VRE) [17,18,19], which positions lanthipeptides as promising antibiotics.

The genetic basis for lanthipeptide biosynthesis resides in specific genes located within biosynthetic gene clusters (BGCs). These BGCs typically include at least the precursor gene *lanA* and the biosynthetic enzyme gene *lanM*. The presence of additional components, such as the genes that encode transporter proteins or specialized proteases, varies across BGCs [20,21]. Notably, some class II precursor peptides contain a double-glycine motif within the leader peptide, which serves as a proteolytic cleavage site [22,23,24,25]. Furthermore, BGCs expression is controlled by specific pathways that involve activation of various regulatory protein families, including two-component systems (TCS) and *Streptomyces* Antibiotic Regulatory Proteins (SARP) [19,26,27].

BGCs of class II lanthipeptides occur in *Bacillus* spp. and *Lactobacillus* species, as well as marine cyanobacteria from the genera *Prochlorococcus* and *Synechococcus*, and have recently been detected in the domain Archaea [3,19,28,29,30]. Actinobacterial genomes represent a substantial reservoir of class II lanthipeptides [31,32]. Among these, actagardine and cinnamycin are the most notable members described in Actinomycetota. Actagardine is a globular 19-amino-acid peptide with one Lan ring, three MeLan rings, and a unique sulfoxide group, introduced by a luciferase-like monooxygenase enzyme. Actagardine demonstrates selective antibacterial activity against the multidrug-resistant *Clostridioides difficile* [33,34,35]. Cinnamycin family members feature a 19-amino-acid globular peptide with one Lan ring and one MeLan ring, a C-terminal lysine-alanine linkage, and a hydroxylated aspartate [4,9,27]. Notably, certain peptides, such as cinnamycin B and duramycin, bind non-specifically to phosphatidylethanolamine (PE), a membrane component in prokaryotic and eukaryotic cells, thereby supporting their potential as molecular markers in anticancer therapies [36,37,38]. Both the actagardine and cinnamycin families have conserved BGC structures and highly similar LanM sequences across producing organisms. However, information on other class II lanthipeptide families remains scarce.

Genome mining offers an alternative approach to discovering novel class II lanthipeptides with biotechnological and pharmaceutical potential. A study by Ayikpoe and colleagues [39] identified a new class II lanthipeptide group featuring a DUF6229 within the precursor peptide. The lanthipeptide LanII encoded within the genome of *Dyella* sp. 333MFSha, containing one Lan and two MeLan rings, showed antibacterial activity against *Klebsiella pneumoniae*. The study also uncovered orthologous peptide sequences in genomes from the family Rhodanobacteraceae, termed rhodanocins. Recent work by Bierman and Walker [40] identified precursor peptides containing the DUF6229 domain within the genomes from *Lysobacter* spp., that possess rhodanocins-like features. Previously, a bioinformatic study identified a group of five DUF6229-containing sequences, though their BGCs remained poorly characterized [41]. In this context, the aim of this study was to analyze lanthipeptide biosynthetic clusters in actinobacterial genomes that encode DUF6229-domain peptides, as identified in various databases. We established relationships among these peptides based on their leader peptide sequences and propose naming this new lanthipeptide family actinopeptins.

## 2. Results

### 2.1. Prevalence of the DUF6229-Containing Peptides

**Figure 1:**
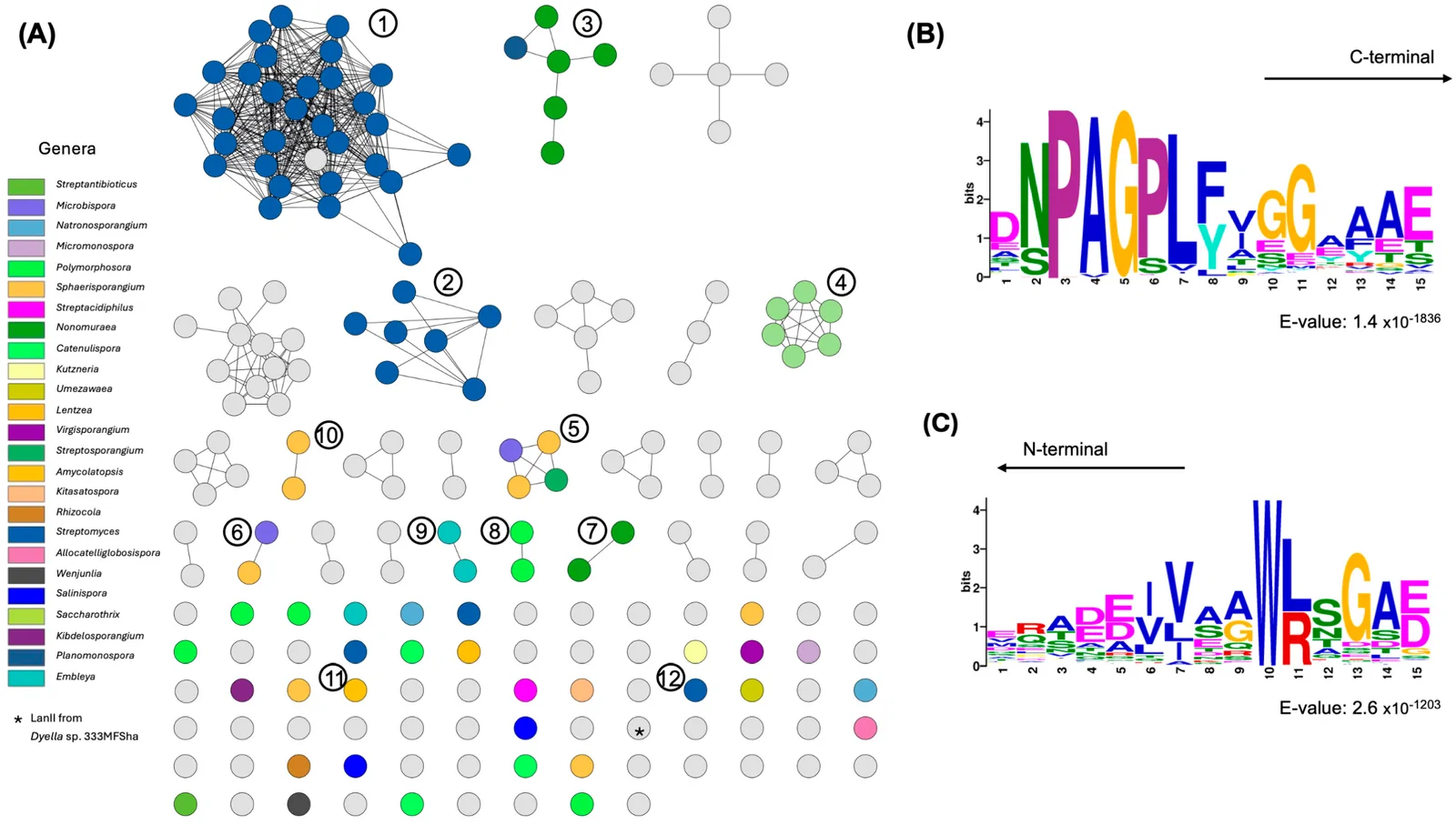
(**A**) Sequence Similarity Network (SSN) of the sequences recovered from InterPro containing the PF19740 (DUF6229) domain. Sequence nodes of the genera belonging to the phylum Actinomycetota are shown in different colors and each cluster is represented by a specific number (from 1 to 12). Additionally, the peptide LanII and the assignment number for each cluster from Actinomycetota are indicated according to the EFI-EST analysis. Sequences from other phyla are colored gray. (**B**) Conserved motif located at the C-terminal side of the leader peptide segment with the lowest E-value. (**C**) Conserved motif at the N-terminal side of the leader peptide. E-values are indicated in the figure caption.

A bioinformatic search for the DUF6229-containing peptides was conducted across several databases. Searching the UniProt database yielded 231 entries classified with the PF19740 (DUF6229) domain. In contrast, a search for the term ‘DUF6229’ returned 1153 protein entries in the NCBI database, many of which were categorized as multispecies results. We performed a sequence similarity network (SSN) analysis using the PF19740 domain as input, as shown in Figure 1A. In this analysis, each node from the genera within the phylum Actinomycetota is colored. As anticipated, the DUF6229 sequences were found in the genomes from four different phyla: Actinomycetota, Pseudomonadota, Myxococcota, and Verrucomicrobiota, with sequences of the phylum Actinomycetota being the most prevalent. The SSN consists of 33 clusters and 68 singletons, although only 12 clusters and 41 singletons are from the genomes of Actinomycetota. The previously characterized LanII belongs to the family Rhodanobacteraceae and is represented in Cluster 29, which merged with a sequence from *Luteibacter* sp. 22Crub2.1. Cluster 1, the largest, contains 41 sequences from strains of the genus *Streptomyces*, and one from *Actinobacteria bacterium* OK006, distributed across 31 nodes.

Based on this analysis, we obtained a genome neighborhood network (GNN) in which proteins with the DUF4135 domain were observed in each cluster. This corresponds to the dehydration domain of the biosynthetic enzyme LanM, followed by proteins belonging to the ATP-binding cassette (ABC) transporter family. Furthermore, we identified the [NS]P[AV]G[PDES] motif among the sequences from InterPro (see Figure 1B). This motif is conserved on the amino-terminal side (around positions 15–30), corresponding to the leader peptide region. This section is highly conserved among lanthipeptides from the same family to ensure the correct recognition and installation of post-translational enzymatic modifications by biosynthetic enzymes. Its presence in these sequences highlights its importance in binding or interacting with the lanthipeptide synthetase LanM. This result is consistent with the conservation of the motif found in sequences retrieved from the NCBI database, where the position side to proline can be any other amino acid. To identify these peptides, we will refer to this motif as XP[AV]GX.

Additionally, in both cases we identified an X[VLIA]XXW[LR]X motif on the N-terminal side of the leader peptide (Figure 1C). The accession numbers of the sequences from the genomes of the phylum Actinomycetota included in this analysis are provided in Supplementary Table S1.

In class II lanthipeptides, researchers have speculated on the different catalytic motifs or binding sites in the leader peptide required for proper peptide modification by LanM [42,43]. These studies, primarily conducted in Firmicutes, report a prevalence of glutamic acid (E) and aspartic acid (D). Notably, all leader peptide sequences consistently feature a high presence of E, typically located at the C-terminal end, particularly when the XP[AV]GX motif is identified. Interestingly, proline (P) and glycine (G) are the only conserved amino acids in the motif. Proline is an amino acid that restricts mobility and is rarely found in the leader peptides of lanthipeptides. In class I lanthipeptides, proline is a highly conserved amino acid in the −2 position (with respect to the cleavage site) and plays a crucial role in recognition by the LanBCT biosynthetic complex [5,42]. However, in class II lanthipeptides, proline appears in mersacidin-like lanthipeptides, which are produced by various strains of the genus *Bacillus*, although the function of this amino acid in the biosynthesis of mersacidin remains unclear. Further studies have shown that introducing proline into the leader peptide of lacticin 481 affects its binding to its biosynthetic enzyme LctM, due to the loss of the α-helix structure, which is important for this interaction [44]. In this context, proline is also conserved in the leader peptide of LanII peptide, which is processed correctly by its LanM [39].

### 2.2. RiPPs Containing the DUF6229 Domain Form a New Family of Lanthipeptides

Peptides possessing the DUF6229 domain were examined to determine whether they could be classified within the cinnamycin family. However, the motifs found in the DUF6229 sequences are not present in the leader peptide of that family. We detected the motif XP[AV]GX in peptides such as mersacidin; in fact, some of the DUF6229 sequences are labeled as “mersacidin/lichenicidin peptides”. An inspection of the sequence logos, however, reveals several differences between the leader and core peptides and those of the mersacidin group. To verify that the DUF6229 group does not belong to the mersacidin-like peptides family and to ensure that they are distinguished from the cinnamycin and actagardine family, we aligned the amino acid sequences of LanA and LanM from various origins. To ensure that all sequences correspond to class II lanthipeptides and avoid false positives, we downloaded the complete genomic DNA sequences of strains containing DUF6229-containing peptides. After analyzing all the selected genomes with antiSMASH, we recovered the LanA and LanM sequences.

All the LanM sequences share a high percentage of identity (ranging from 50% to over 80%) within the same genus. The main motifs reported in the dehydration and cyclization domains were localized and verified; a glutamine residue at position 598 of the dehydration domain, reported in the LanMs from clusters of *Bacillus* spp., was absent, and instead, a glutamic or aspartic acid was identified in its place. A previous study showed that this amino acid is important for releasing the dehydrated peptide from the active site [45]. Although substituting glutamine for alanine prolonged peptide production from 15 min to 2 h, the replacement did not eliminate enzyme activity. Furthermore, the LanM enzyme from the BGC of *Dyella* sp. 333MFSha preserves this change in its sequence, and the study by Ayikpoe and collaborators [39] shows that the LanII peptide is fully modified, indicating that this mutation preserves LanM’s catalytic activity. Additionally, we did not detect the ProcM-like motif (CCG) within the cyclization domain; this motif is associated with promiscuity in synthetases from cyanobacterial strains; instead, the classic CHG motif is present (Supplementary Figure S1). However, it is worth noting that the LanM sequences from clusters of *Streptomyces* sp. RLB133 and *Kutzneria chonburiensis* SMC256 are shorter than other sequences in the group. The sequence alignment showed that both sequences lack the conserved motifs in the potential cyclase domain.

**Figure 2:**
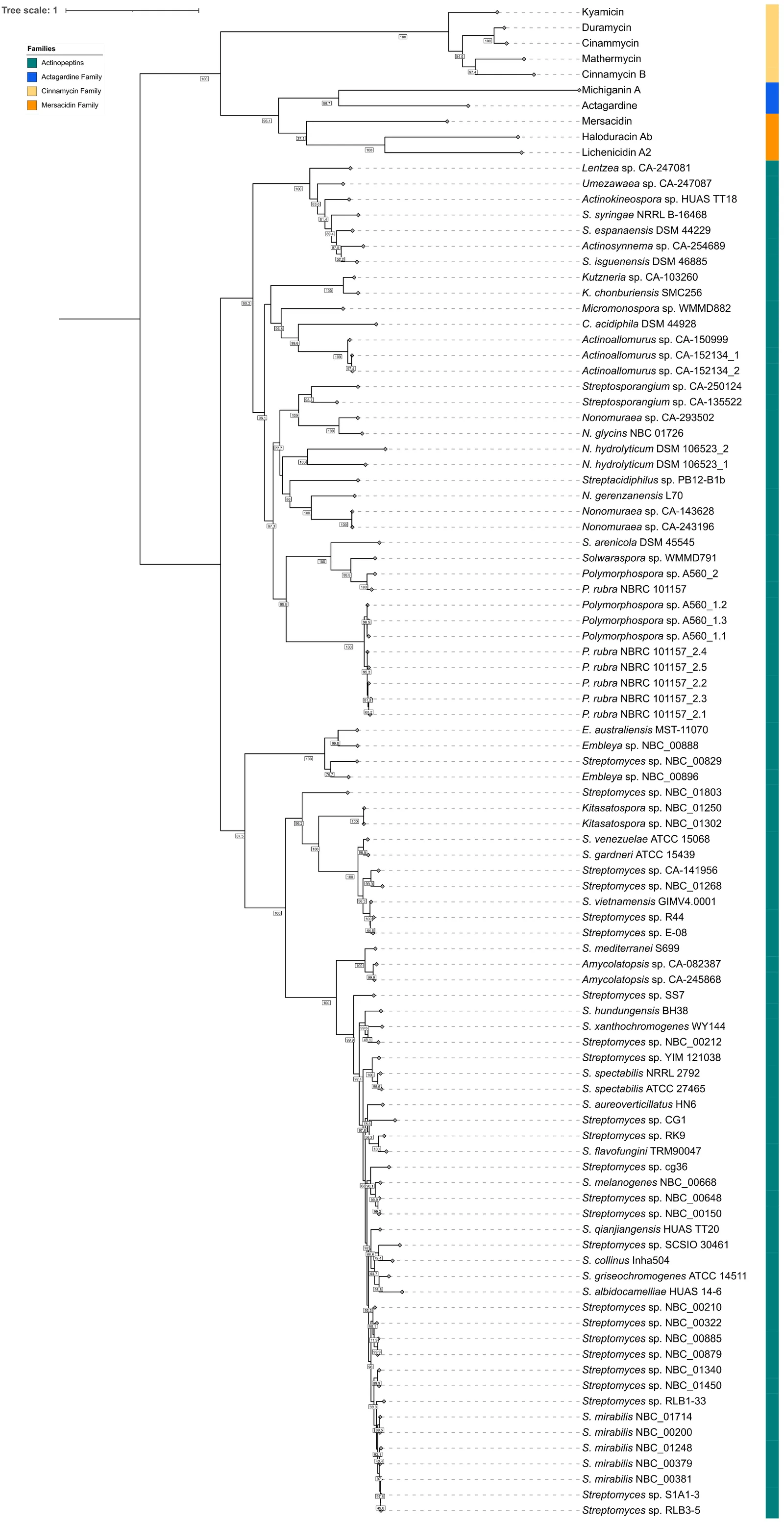
Phylogenetic tree of LanA and LanM sequences from the actinopeptin family. The tree marks the separation of the actinopeptin family from the mersacidin, actagardine, and cinnamycin families. Data for this analysis were obtained from antiSMASH v8.0, and the phylogenetic tree was generated using IQ-Tree within 1000 bootstrap replicates using the Q.PFAM+F+I+R5 model. Bootstrap values for each branch are represented.

A phylogenetic analysis was performed using the previously generated multiple sequence alignment. The resulting phylogenetic tree (Figure 2) showed that the actagardine-like, cinnamycin-like, and mersacidin-like peptides are grouped into a different clade from the DUF6229-containing peptides; indeed, the other families had longer branches than the other members of the DUF6229 group. This result suggests divergence among the mersacidin, actagardine, cinnamycin and DUF6229 families, while also indicating a common ancestor, since they are clustered as class II lanthipeptides. In this same analysis, we also noted that the mersacidin and actagardine families form a clade, while the cinnamycin-like peptides are localized in another clade, suggesting a divergent process between the two families.

With respect to DUF6229 peptides, it highlights the formation of two different clades, one of which includes the most sequences from genomes of the genus *Streptomyces* and the other includes sequences from “rare actinobacteria”. This result could reflect differences in the sequences of both LanA peptides and LanM proteins, which are a result of differences in the evolutionary process, despite both belonging to the same phylum; in fact, the best model used to construct the phylogenetic tree could reflect adaptive evolution of each biosynthetic gene. We compared LanA and LanM and created a heatmap of identity (see Supplementary Figure S2). In this analysis, we observed that the identity between the actinopeptins and the other class II lanthipeptides is lower than 30%.

In addition, a comparison between the BGC dataset and the MIBiG 4.0 repository (which includes clusters of other class II lanthipeptide families) showed no clustering. Based on these results, we propose that these are distinct lanthipeptide families that we named actinopeptins. However, the conservation of amino acids of the XP[AV]GX and X[VLIA]XXW[LR]X motifs in the leader peptide underscores their importance for binding or LanM recognition.

### 2.3. Genomic Context of Actinopeptin BGCs

We analyzed the genomic context of the recovered BGCs, based on the antiSMASH results. Many lanthipeptide clusters are bioinformatically identified as neighboring hybrid clusters, implying the co-localization of other core biosynthetic genes near *lanA* and *lanM*, which contain genes for terpenoid biosynthesis, non-ribosomal peptide synthetases (NRPS), and other peptide production systems such as non-alpha poly-amino acids (NAPAA), azole-containing ribosomally synthesized peptides or even clusters of other lanthipeptides. It is important to mention that the production of hybrid lanthipeptides has been reported in other systems (such as lipolanthines) [46], but it is intricate to predict any specific biosynthetic pathways to define a hybrid product of this family. Notably, more than 50% of BGC from the analyzed genomes are classified as class II lanthipeptide clusters not associated with any other biosynthetic gene clusters.

To analyze the formation of subfamilies within the actinopeptin family, we generated a gene cluster family (GCF) in BiG-SCAPE using the same dataset used to construct the phylogenetic tree. The analysis, with a cutoff of 0.3 (the most restricted threshold), identified 12 GCFs and 56 singletons (see Supplementary Figure S3). As the cutoff increases, more families tend to group together. However, this depends on the genus rather than on the similarity of the clusters, implying differences in the tailoring enzymes and regulatory proteins associated with the key biosynthetic proteins LanA and LanM. These differences could result in a product with different characteristics due to a different biosynthetic pathway. Thus, we selected the cutoff 0.3, where we observed a high similarity in the distribution of proteins throughout the BCG. The largest number of BGCs in a family was five, while other families had three or two members. Ten clusters were formed for clusters from strains of the genus *Streptomyces*, one of *Polymorphospora*, one of *Nonomuraea,* and the last for the genus *Amycolatopsis* (see Supplementary Table S2). Notably, in family A, four out of the five members were isolated from microscale soil grain samples [47]. Additionally, all other GCFs are formed of clusters that come from strains from unspecified species, except for those of *S. venezuelae* ATCC 15068 and *S. gardneri* ATCC 15439. The latter was considered a strain of *S. venezuelae* until its recent reclassification [48].

A detailed overview of each analyzed subfamily is provided in Supplementary Table S2. Additionally, using the BiG-SCAPE Query function, we do not observe any clustering of the mersacidin BGC with any actinopeptin cluster.

### 2.4. Classification of Sequences of the Actinopeptin Family

We noted a high diversity among the analyzed BGCs, making it difficult to classify actinopeptins into distinct groups based on their cluster organization. To address this, we examined the clades formed in the phylogenetic tree corresponding to specific variations in the precursor peptide, particularly in the core segment, as well as the identity of LanM enzymes. The identity heatmap reveals that identity values reached up to 99% in sequences from the genus *Streptomyces*. In sequences from other genera, such as *Embleya*, the identity ranges from 44% to 97%. These differences grouped the sequences into distinct clades.

To analyze these punctual differences, we conducted a “motif search” on the precursor peptide sequences to identify the possible cleavage sites. While the exact cleavage site is not always clear, we observed a particular prevalence of amino acids at the double-glycine motif (GG), which separates the two peptide segments. Furthermore, this search revealed significant variability at the carboxyl-terminal end. Notably, two cysteines at the carboxyl-terminal end are conserved in most cases. Ayikpoe and colleagues [39] predicted differences in the number of possible Lan/MeLan rings between rhodanocins and lanthipeptides from the phylum Actinomycetota, because the latter contains an additional cysteine. However, we observed several potential cyclization patterns, depending on the number of cysteine residues within the core peptide. For instance, cluster 1 of the SSN, which comprises sequences of the genus *Streptomyces*, contains only three cysteines following the GG motif. In contrast, cluster 21, consisting of sequences of the genus *Embleya*, contains four cysteines. This difference is consistent with slight variations in the cleavage site. However, we do not rule out the possibility of another cleavage site located upstream of the proposed motif. This speculation arises from the presence of a region with a low percentage of threonine and serine residues between the GG site and the first ring-forming amino acid, resembling the cleavage sites found in mersacidin and actagardine [22,34].

**Figure 3:**
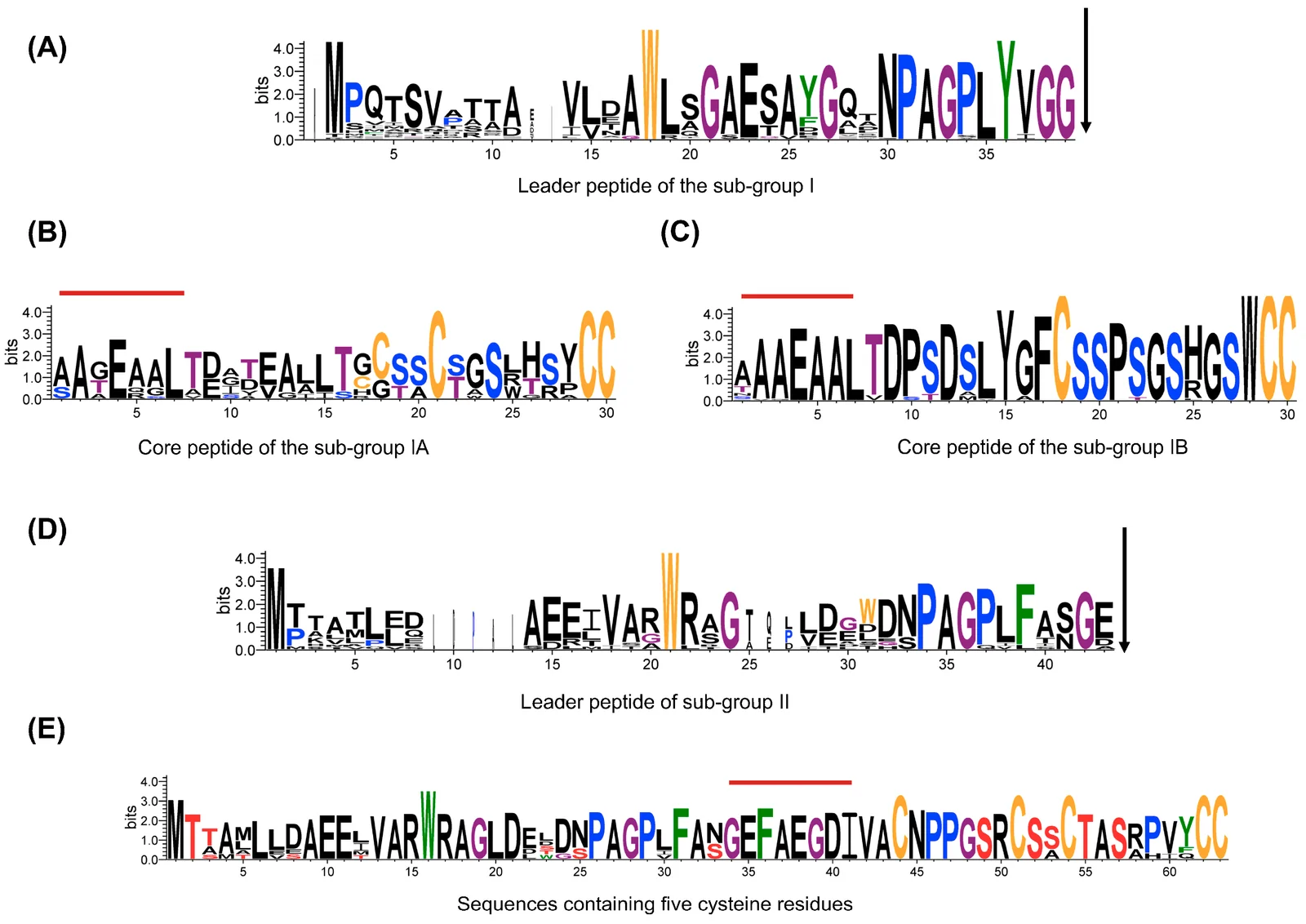
Sequence logos of the leader and core peptides from different subgroups of the actinopeptins family. (**A**) The leader peptide of sequences of the subgroup I contains three amino acids located between the motif XP[AV]GX and the double-glycine site (indicated by the black arrow). (**B**,**C**) Core peptides from subgroups IA and IB, respectively. In both cases, a red segment indicates regions with low conservation of ring-forming amino acids. (**D**) The leader peptide of sequences belonging to subgroup II, where the proposed cleavage site occurs after a Gx site (indicated by the black arrow). (**E**) The sequence logo representing sequences that contain five cysteine residues, from rare actinobacteria.

Here we propose an alternative classification based on the identity between both LanA and LanM sequences, which merge within their organization within the phylogenetic tree and the specific features localized into the precursor peptide (see Figure 2 and Figure 3). Nevertheless, the following classification clarifies the distribution of sequences observed across all previous analyses (see Supplementary Table S3). Table 1 shows the main characteristics used to classify sequences belonging to the actinopeptin family.

#### 2.4.1. Group I

In this group, a double-glycine motif (GG) marks the beginning of the core peptide, with both GG and XP[AV]GX motifs separated by three amino acids (see Table 1). Highlights the presence of nonpolar and uncharged amino acids in this segment (Figure 3A).

This group can be further divided into two subgroups based on the number of cysteine residues that form rings. Subgroup IA contains lanthipeptides with four rings (see Figure 3B). This includes peptides from genera such as *Kitasatospora* and *Embleya*, as well as some strains of *Streptomyces* including *S. gardneri* ATCC 15439, *S. venezuelae* ATCC 15068, and *S. vietnamensis* GIMV4.0001. Within the phylogenetic tree, the clade comprising *Kitasatospora* and *Streptomyces* has peptides with two serines between these cysteines; in contrast, the *Embleya* clade has sequences containing three different amino acids at this position. Additionally, the sequences from the genus *Streptacidiphilus* and *Nonomuraea gerenzanensis* L70 share the same cysteine distribution but are longer than the other sequences in this group. In this classification, the biosynthetic gene clusters associated with *Micromonospora, Salinispora,* and *Catenulispora* are assigned to this subgroup. However, specific differences at the N-terminus of the LanA indicate that they belong to a different clade within the phylogenetic tree.

The subgroup IB comprises sequences that contain only three cysteines in the core peptide (see Figure 3C). This subgroup primarily consists of peptides from unclassified strains of *Streptomyces*, which are grouped within the same clade on the phylogenetic tree.

In contrast to group 1A, the position of the three cysteines in all analyzed sequences within group IB is conserved, as illustrated in Figure 3C. Notably, serine is highly represented between the first and second cysteine residues. However, the grouping of these sequences within the phylogenetic tree suggests an early evolution of subgroup IB, supporting that these sequences may have lost a cysteine residue. However, further studies are needed to confirm this hypothesis.

#### 2.4.2. Group II

This category includes sequences that lack a double-glycine motif. In these peptides, the cleavage site is not well defined, but similarities among most amino acids suggest a potential motif (see Figure 3D). The number of residues separating the XP[AV]GX motif from the putative cleavage site can vary from three to four, with the conservation of aliphatic and aromatic residues.

In most cases, position −2 is occupied by a conserved glycine, while position −1 can be a variable amino acid (see Table 1). The clade formed by sequences containing five cysteines includes rare actinobacteria such as *Umezawaea, Lentzea,* and *Saccharothrix*. Notably, sequences in this clade feature another “Gx” motif located upstream of the first putative cleavage site, and this motif is also followed by a charged amino acid adjacent to the glycine residue (see Figure 3E). Furthermore, none of the ring-forming amino acids are located between the two putative cleavage sites, suggesting this region could represent another starting point for the core peptide.

#### 2.4.3. Unclassified Sequences

We observed in the phylogenetic tree that some sequences do not fully cluster into defined clades, primarily due to variations in their cleavage sites. Analysis of their BGCs revealed that many strains encode a specific protease near *lanA* and *lanM*, primarily a metallopeptidase. The presence of these proteins may explain the variations in cleavage sites and their positions in the phylogenetic tree. Additionally, these sequences form singletons in both SSN and BiG-SCAPE analyses. Included in this group are peptides from genera such as *Kutzneria*, *Actinoallomurus*, and *Nonomuraea* sp. C-293502.

However, we recognized that the limited sequence representation within this clade complicates the analysis, resulting in poorly supported clades in the phylogenetic tree. A mix of this group, group II, and the multicomponent group are present in the analysis. Therefore, it is essential to consider a broader diversity in these sequences, especially since they originate from genomes of strains what are termed “rare actinobacteria”. It is likely that more sequences will become available in the future, leading to a refined classification.

#### 2.4.4. Multicomponent Lanthipeptides Group

The actinopeptins family exhibits diversity, including some BGCs that contain different precursor peptides, either within the same cluster or in other regions of the genome. To date, we have identified three genera that display this characteristic: *Natronosporangium hydrolyticum* DSM 106523, *Actinoallomurus* sp. CA-152134, and two distinct strains of *Polymorphospora*.

**Figure 4:**
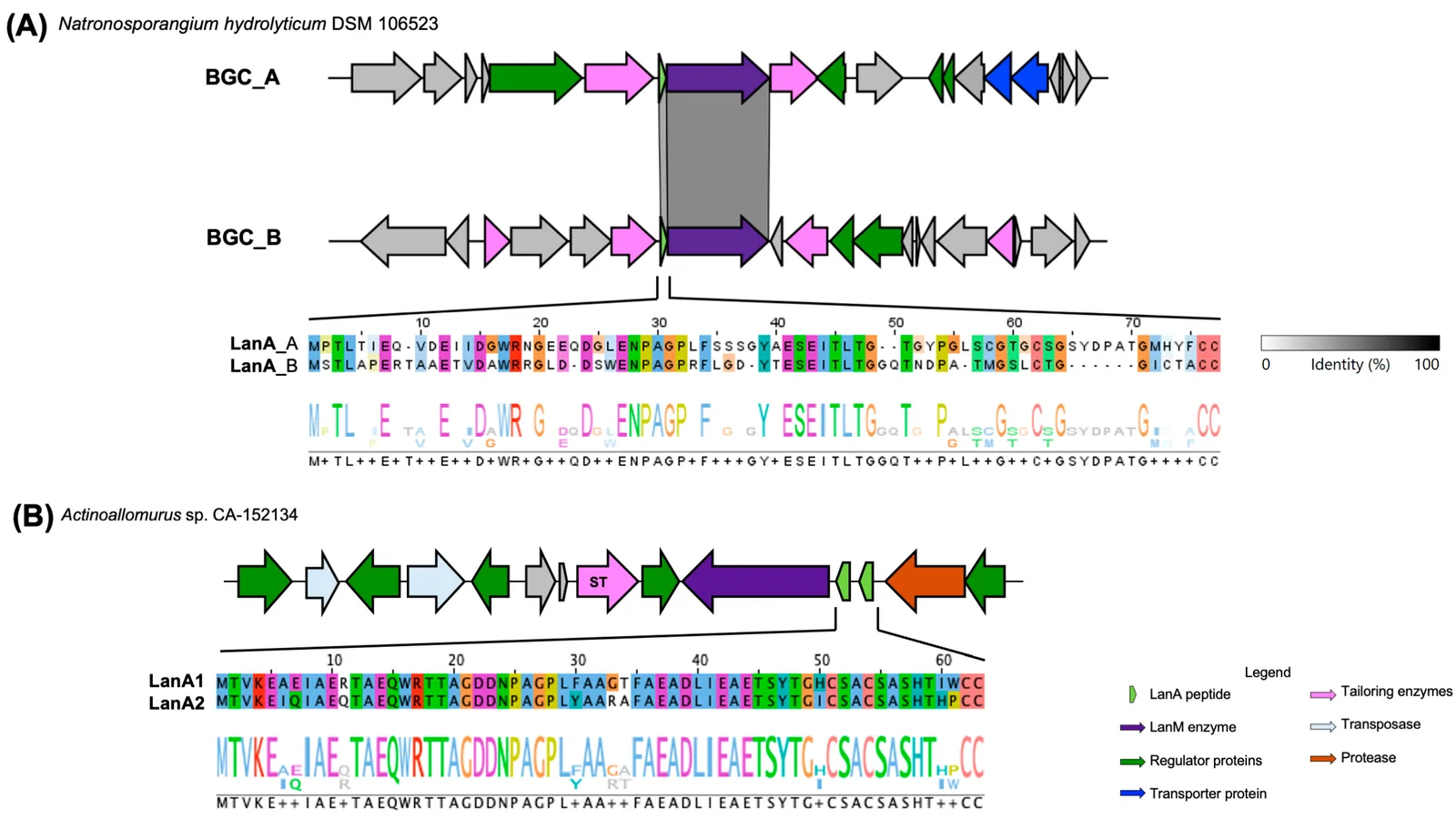
Multicomponent lanthipeptides in the genomes of rare actinobacteria. (**A**) Comparison between the BGCs of *N. hydrolyticum* DSM 106523 is shown. Despite their similar organization, the identity percentage between the two clusters is low. Notably, the alignment of the two precursor peptides reveals a high level of conservation in the leader peptide, while the core peptides exhibit significant differences. (**B**) Two-component lanthipeptide from *Actinoallomurus* sp. CA-152134. The composition of both peptides is conserved with regard to the ring-forming amino acids. It is possible that these lanthipeptides originated from a duplication process. ST: sulfotransferase.

*Natronosporangium hydrolyticum* DSM 106523, isolated from saline soda soil [49], possesses two class II lanthipeptide BGCs belonging to the actinopeptin family (Figure 4A). Initially, we suspected this might be due to a duplication process, but the differences between the two clusters suggest they produce distinct peptides. The LanA peptides share 44.2% identity. However, predicting the cleavage site is challenging because the double-glycine motif is absent. The high prevalence of serine and threonine residues downstream of the XP[AV]GX motif suggests that this site corresponds to ‘GY’ and ‘GD’ in LanA_A and LanA_B, respectively, although a second GG motif is present in LanA_B.

LanA_A peptide is longer than the LanA_B peptide, and their LanM enzymes also share this characteristic. These findings support the co-evolution of peptide and biosynthetic enzymes [5,50]. Additionally, there are other notable differences in their BGCs. The regulatory mechanism in BGC_B is controlled by a two-component system, while BGC_A involves at least three regulatory proteins from the ArsR, SARP, and LuxR families. Furthermore, the only shared tailoring enzyme is a flavin adenine dinucleotide (FAD)-linked oxidase-domain protein, whose gene is located near the core biosynthetic genes, and shows low sequence identity with them (23.1%). BGC_A contains a gene annotated as an acyl-CoA dehydrogenase family protein, primarily described as a key enzyme in fatty acid metabolism.

BGC_B can produce an aminoglycoside phosphotransferase (APH). This class of enzymes is reported to be involved in the dehydration of the precursor peptide of cacaoidin (CaoK and CaoX). This enzyme can participate in the dehydration reaction together with the LanM synthetase. Notably, there are two genes that encode S-adenosylmethionine (SAM)-dependent class I methyltransferases that can install a methyl group into the core peptide. This characteristic also constitutes a tailoring modification in cacaodin, increasing its bioactivity [51]. In the BGC_A, there are three annotated genes as metal ABC transporter permeases. To our knowledge, no such permease transports lanthipeptides out of the cell.

Unlike *N. hydrolyticum*, *Actinoallomurus* sp. CA-152134 encodes two precursor peptides that are processed by a single biosynthetic enzyme (Figure 4B). A comparison of these peptides reveals slight differences in non-ring-forming amino acids and a high degree of amino acid conservation in the leader peptide. Notably, there is no evident cleavage site in either sequence. Only a ‘GT’ motif was identified in LanA_A. Additionally, a metallopeptidase of the M56 family is encoded in the BGC and may be responsible for proteolysis. These peptidases are common in class III lanthipeptides, although, in some cases, they are found outside the BGC [52,53]. Furthermore, a sulfotransferase (ST) gene is present. Recently, two new sulfonated lanthipeptides, SslA and SscA, were identified in *Streptomyces* [54]. This modification occurs at a tyrosine residue present in both precursor peptides; in fact, the SscA peptide shares the XP[AV]GX motif, but with notable differences in the core peptide, including the position of the modifiable tyrosine. However, a cluster comparison reveals low sequence identity between the two BGCs; even the sulfotransferases sequences share only 17.7% identity with the ST (WP_073758 105.1) from the Ssc cluster, which is a lower percentage than the 57.0% identity among the STs from both sulfonated peptides [54]. Additionally, there is a notable absence of PAPS synthetases; the sulfuryl donor molecule was observed in *Actinoallomurus*, suggesting a different biosynthetic pathway for both BGCs and supporting the idea that they belong to a different family of lanthipeptides. *Actinoallomurus* sp. CA-150999 produces a sulfotransferase with similar characteristics. An analysis of the regulatory sequence shows that *lanM* and *lanA_A* form an operon.

**Figure 5:**
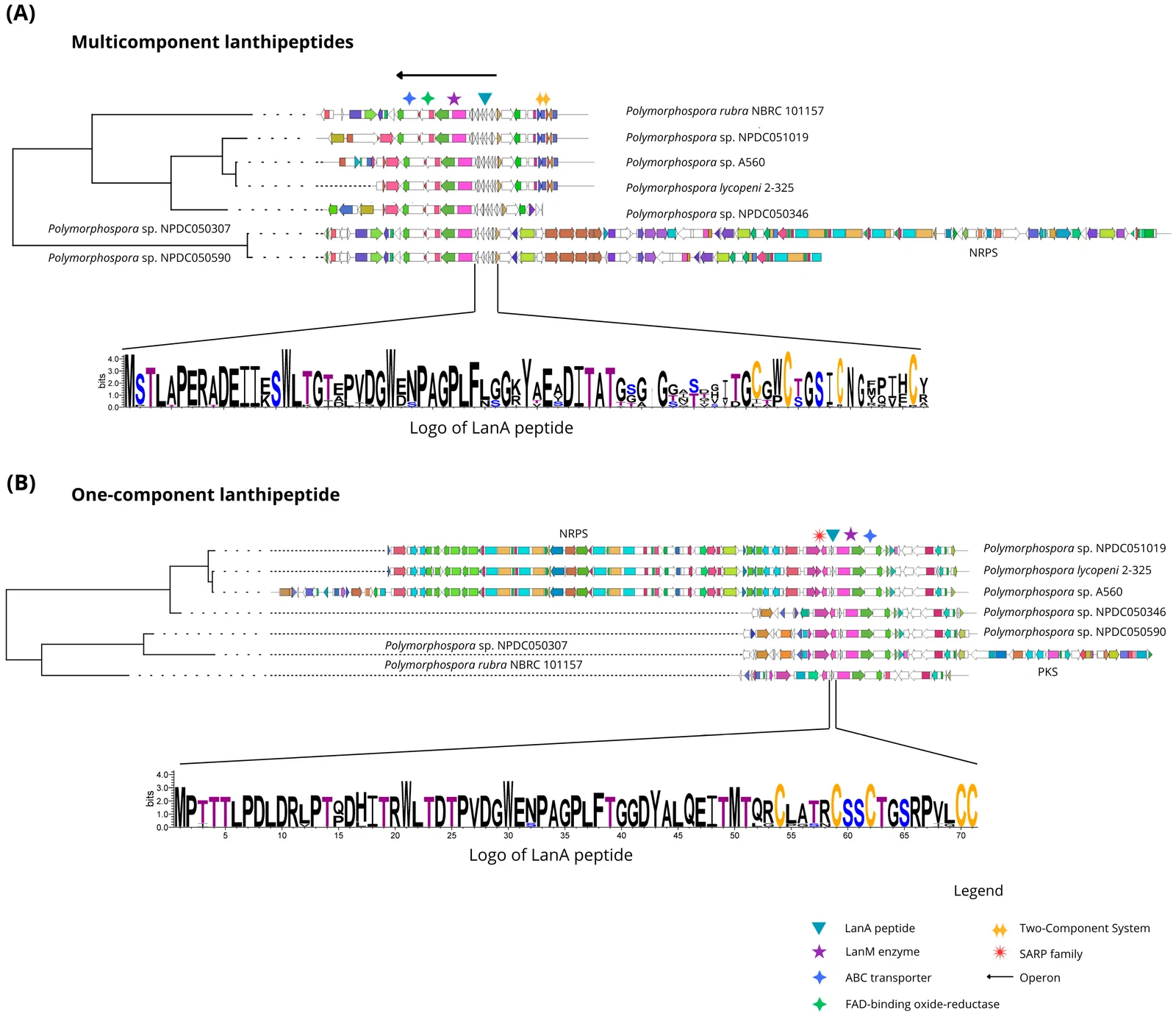
Families of class II lanthipeptides in *Polymorphospora* genomes. (**A**) BGC of multicomponent lanthipeptides. The divergence among the clusters and the LanA peptide logo are shown. In general, the biosynthetic pathway is conserved, including the tailoring modification (FAD-containing domain protein) and a probable two-component system involved in regulation. (**B**) One-component BGC. In this case, half of the clusters are hybrids with an NRPS BGC or a PKS BGC in *Polymorphospora* sp. NPDC050307. As shown by the precursor peptide logo, unlike multicomponent clusters, these lanthipeptides conserve a double-glycine motif and two cysteines at the C-terminus in both the leader and core peptides, respectively. In both cases, the color in the BGC indicates the motif recognized within the genes.

*Polymorphospora* sp. stands out within the actinopeptin family due to its characteristics. Two different BGCs are encoded in both *Polymorphospora* strains included in this study. In both strains, one cluster corresponds to a multicomponent lanthipeptide. In *Polymorphospora* sp. A560 encodes three distinct precursor peptides, whereas in *P. rubra* NBRC 101157, it can produce five distinct peptides containing the DUF6229 domain. In the NBCI database, the two strains are the only ones with the ‘complete’ genomes. To provide more information, we include the other five available genomes to determine whether these features are common characteristics within the genus, and we perform a BiG-SCAPE analysis (Figure 5). As expected, we observed clustering with only one peptide precursor, and, at a lower cutoff, clustering of more than one peptide. However, some clusters merged with other biosynthetic clusters, such as NRPS and polyketide synthases (PKS). It is important to emphasize that in the ‘complete’ genomes, the multicomponent lanthipeptides do not hybridize with other clusters. Furthermore, unlike other actinopeptins, the multicomponent lanthipeptides exhibit distinct characteristics in both the leader and the core peptides (Figure 5A). In general, the leader peptide maintains high identity, and most of them share a double-glycine site after the XP[AV]GX motif (see Figure 5A). All clusters analyzed contain at least two Group 1 sequences and one sequence with the possible ‘GR’ motif as a cleavage site. Some peptides have a glycine domain consisting of two to five amino acids within the putative core peptide. In all cases, these peptides lack the double-cysteine motif at the C-terminus, and the number of cysteines ranges from three to four. Intriguingly, some strains such as *P. rubra* NBRC 101157 encoded an incomplete peptide that apparently retains only the leader peptide, followed by a truncated core. Additionally, on the side of *lanM* there are genes encoding a FAD-linked oxidase, an ABC transporter, and the precursor peptides; these sequences form an operon, suggesting the presence of a complete lanthipeptide production system. In fact, a two-component system (TCS) may be responsible for the pathway regulation in this multicomponent cluster.

The second BGC contains only one precursor peptide and, like the multicomponent BGC, conserves the cluster organization (Figure 5B). These clusters contain the *lanA*, *lanM,* and *lanT* (ABC transporter) genes organized into an operon. On the other side of the *lanA* gene, a SARP family transcriptional regulator gene is present, suggesting that the peptides may have biological activity as antibacterial compounds. Interestingly, the BGC from the *P. rubra* NBRC 101157 strain is the only one to contain a gene encoding a protein with an FAD-linked oxidase domain within the same operon. The results of both phylogenetic analyses clearly indicate that the clusters of the strain *P. rubra* NBRC 101157 are divergent from those of other members of the genus. It is worth noting that this strain was isolated from soil around mangrove roots [55]. As observed, *Polymorphospora* sp. NPDC051019, *P. lycopeni* 2-325 and *Polymorphospora* sp. A560 shares a hybrid cluster between the lanthipeptide and an NRPS. The leader peptide of these peptides shares characteristics with Group 1; only a few slight differences among them are localized in the leader peptide, as well as in the core peptide, where the position of ring-forming amino acids is conserved. The double-cysteine motif is located on the carboxyl-terminal end and can potentially form five rings. It is noteworthy that this “one-peptide cluster” is closely related to other actinopeptin clusters, and that the multicomponent cluster may have arisen from speciation within the strains, but further analysis is necessary to corroborate this hypothesis.

### 2.5. Rare Proteins Associated with BGCs

GNN analysis revealed the presence of various enzymatic groups associated with LanA peptide (Supplementary Figure S4). Each group within the family shares some tailoring enzymes. These proteins may be adding some specific functional groups to lanthipeptides after ring installation, although there is no direct relationship between the leader peptide’s characteristics and the presence of specific tailoring enzymes in the actinopeptins family. For example, we observed differences in the prevalence of oxidoreductase enzymes across clusters. FAD-containing oxidoreductases are common enzymes in BGCs from rare actinobacteria such as *Nonomuraea*, *Micromonospora*, *Polymorphospora*, and *Embleya*, and are even found in some strains of *Streptomyces*. However, species of *Nonomuraea*, such as *N. gerenzanensis* L70, belong to subgroup IA, while *N.* sp. CA-243196 (and most of this genus) are classified in group II. In this regard, other oxidoreductases, such as cytochrome P450 (CYP450), are encoded in clusters in *Embleya* and in *Kutzneria* strains.

Methyltransferases are another common class of tailoring enzymes in the actinopeptin family. Some BGCs from the genera *Streptomyces* and *Saccharothrix* encode this enzyme class. Additionally, we observed the presence of short-chain dehydrogenase/reductase (SDR) proteins (PF00106). This enzyme (ElxO) catalyzes the reduction of the pyruvyl-to-lactyl motif in the biosynthesis of epilacin 15x [56]. Several reductases of this family are mainly encoded in the clusters of the genus *Streptomyces*.

Furthermore, the GCF analysis revealed that in families composed of *Streptomyces* spp. and *S. mirabilis*, a stage II sporulation protein E (SpoIIE) gene is largely conserved adjacent to the *lanM* gene. This protein is involved in the sporulation process in *Bacillus* spp. [57,58]; however, in the phylum Actinomycetota, this process is regulated by other systems [59,60].

Although protease absence is common among actinobacterial clusters, we observed that some BGCs contain proteases of various families, including annotated genes as primary metal-binding proteases located near the *lanM* and *lanA* genes, as previously discussed.

The GNN analysis reveals the presence of the IS200 transposase family and an IS200-like transposase in cluster 12, as well as two transposase DDE domains in *Streptomyces* sp. HUCO-GS316 and *S. luteolifulvus*. Furthermore, members of subgroup I such as *S. mirabilis* NBC_00200 and *S. vietnamensis* GIMV4.0001, have different transposase families encoded in their BGC. The multicomponent clusters contain transposase genes from different families, such as the IS30 family in *Actinoallomurus* sp. CA-152134, while *P. rubra* NBRC 101157 contains a gene encoding an RNA-guided endonuclease InsQ/TnpB family protein side to biosynthetic core genes.

## 3. Discussion

In this work, we analyzed the prevalence of the actinopeptin family within the phylum Actinomycetota, focusing on 85 genomes that contain at least one sequence annotated as a peptide containing the DUF6229 domain. Although a few examples had been reported previously [41], the information has not been updated. In the present work, we demonstrated that the biosynthetic pathways associated with this family are highly diverse.

Comparative studies demonstrated the presence of a well-conserved motif at the leader peptide. This motif is similar to those reported in other class II lanthipeptides, such as those belonging to the mersacidin and actagardine families, and in other two-component lanthipeptides, such as haloduracin and lichenicidin, demonstrating the highly conserved nature of this region since the divergence of the class II lanthipeptides. Nevertheless, the main difference between the actinopeptin family and both groups lies in the cleavage site and, primarily, in the core peptide. Specifically, the absence of both the mersacidin-like lipid II-binding motif and a processing mechanism similar to mersacidin highlights its novelty. The high number of singletons in the SSN of precursor peptides reflects the high variability among the core peptides; yet this study utilized a classification based on the similarity of the complete precursor peptide. However, to better understand the processing of precursor peptides, it is necessary to produce lanthipeptides in the producer strains to account for possible non-clustering proteases involved in the biosynthetic pathway.

The heterogeneity within this family is also reflected in the biosynthetic pathway, as the specific enzymes are shared only among members of the same genus. This could result from horizontal gene transfer (HGT), which may have facilitated the transmission of BGCs. On this point, there are frequent reports of transposases or other mobile elements flanking class II lanthipeptide BGCs [61]. Furthermore, genome mining analyses have demonstrated the prevalence of mobile elements near class II lanthipeptide BGCs across different phyla, including DDE-domain transposases, which were also present in the genomes analyzed in this study [3,30,62,63]. This characteristic relates to the transfer of the lanthipeptide BGC to other species, either via plasmids or transposons, and may even facilitate their mobility within the same chromosome [64,65,66,67].

The genomes within the phylum Actinomycetota are characterized by their impressive plasticity to transfer genes involved in secondary metabolism in response to environmental signals which involves overcoming barriers among different species for the specific acquisition of genes [68,69]; apparently, clusters of the actinopeptin family are in constant movement across different species, especially in syncretic strains, and this movement also shares the key biosynthetic genes of the same genus, as has been reported in other lanthipeptides clusters such as roseocin and cacaoidin [51,70].

This process could explain why specific BGCs are grouped into certain clusters. Previous studies showed that members of cluster A, as identified in the GCF analysis, harbor many integrative and conjugative elements (ICEs) throughout their entire chromosomes [71]. Specifically, *Streptomyces* sp. S1A1-3 can transfer and receive cargo genes among several sympatric strains; thus, it is not surprising that the class II lanthipeptide BGC may have been transmitted among members of this cluster as part of a diversification of secondary metabolism [72,73,74]. On this basis, Maheshwari and co-workers [41] reported that the evolutionary distances between the cyclase domain of LanM from the actinopeptin family in strains of Actinomycetota are smaller than those between their 16S rRNA sequences, which supports the transfer of this cluster to both bacterial groups.

We did not find a direct relationship between the presence of certain genes in the BGC and their isolation source. However, the low frequency of tailoring enzymes in the analyzed BGCs suggests that their presence may be influenced by strain-specific factors. In fact, multicomponent BGCs may be an example of specialized BGCs. The presence of a protein with an FAD-linked oxidase domain suggests an oxidation-reduction reaction on the precursor peptide, which could be beneficial for the strains.

The presence of multiple LanA peptides, which could result from duplication, is similar to that described for other lanthipeptide systems [75,76]. The high identity percentage among the LanA peptides in these systems, and their position in the phylogenetic tree, suggests that they may have originated through a duplication process. Furthermore, the use of the same biosynthetic enzyme to modify all the peptides reflects the possible promiscuity of this synthetase in response to the bacterial needs, which appears to differ from the Prochlorosins system, but it will be necessary to produce this multicomponent system of lanthipeptides to delimit this feature. In addition, the slight differences in the core peptide among other members of the actinopeptin family and the absence of the double cysteine at the C-terminus support a possible process of specialization. However, information on these “rare actinobacteria” is limited thus far, and it is difficult to draw conclusions based on those characteristics.

Genome mining studies frequently described the co-localization of class II lanthipeptides BGC with proteins specialized in bacterial defense against phages or virulent factors, as well as other specialized proteins such as the acetyltransferases (GNAT) family. However, the rationale behind this remains unclear. It has been established that RiPPs may participate in the defense mechanisms of several bacteria [41,77]. It is worth noting that these enzymes are present across the analyzed BGCs (see Supplementary Table S4). For example, some strains like *Streptomyces* sp. YIM 121038 have many genes associated with their defense and virulence system within their BGC. Additionally, the clusters from the syncretic strains *Streptomyces* sp. S1A1-3 and *Streptomyces* sp. RLB3-5 contain proteins related to anti-phage defense.

The peptide LanII allows us to predict the potential antimicrobial activity of the rodanocins; nevertheless, the widespread distribution of these peptides across various genomes could play a role related to their ecological niche, as was reported for the peptide SapB, which acts as a morphogen peptide in *S. coelicolor* [78], or as defensive signal, as occurs in the microenvironment of the *Synechococcus* production [79]. Additionally, variation in the number of possible rings within each subfamily suggests specialization in target binding, as seen with the cinnamycin group, which has a known affinity for PE, despite being isolated from different sources. However, further studies are necessary to elucidate the specific function of the actinopeptins.

Nevertheless, we do not rule out the possibility that actinopeptins have useful therapeutic properties. The inherent antibacterial activity of class II lanthipeptides could serve as a good indicator for this approach. In general, lanthipeptides from the phylum Actinomycetota exhibit significant antibacterial activity (e.g., microbisporicin and cacoidin) due to highly complex biosynthetic pathways that include multiple tailoring enzymes in their BGCs [80,81]. This study reveals that the BGCs of the actinopeptin family are highly diverse, and some of these differences could be beneficial to secure peptides with improved properties. For example, methylated and oxidized peptides exhibit good antibacterial activity against MDR strains; the presence of a hydroxyl group in cinnamycin-like peptides is relevant to its binding to PE and its potential use in cystic fibrosis [38]. Many of these modifications are installed by tailoring enzymes distributed across the BGCs in our dataset. Furthermore, tailoring modifications can improve certain characteristics of the peptides, such as solubility, a common challenge in the development of lanthipeptides as therapeutic agents. In addition, the presence of distinct ring patterns across the family could confer an advantage in other therapeutic applications, which will need to be verified in future studies.

Finally, we recognized that the diversity of the actinopeptin family will increase with the sequencing of new genomes and the uncovering of new strains from various sources. As this is a genome mining study, the genomic data will drive the design of new strategies to actinopeptins production, and above all, these data will elucidate the functionality of the biosynthetic genes discussed here.

## 4. Materials and Methods

### 4.1. Sequence Similarity Network

The Enzyme Functional Initiative-Enzyme Similarity Tool (EFI-EST) was used to generate the SSN [82,83]. We used the PF19740 (DUF6229) domain as a search term in the “Families” option, with a 30% similarity threshold, to generate the final SSN, with an *E-* value of 5 (≤10^−5^) (last updated on 19 January 2026). The 100% representative node network was visualized in Cytoscape v3.10.4 [84]. The information was manually selected to exclude the non-belonging actinobacteria sequences based on their genus of origin. The colored SSN was used to generate the GNN with a neighborhood size of 10 and a co-occurrence lower limit of 20%.

### 4.2. Selection of Actinobacterial Genomes

A search in the NCBI database was performed using DUF6229 as the query in the “Proteins” option. All sequences from actinobacterial strains were identified, and the corresponding genome sequences were retrieved from the NCBI database. We selected only the genomes in the ‘complete’ category, except for *Salinispora arenicola*, which was highly represented in the search results. For this genus, *S. arenicola* DSM 45545 was chosen because it lacks a complete genome and has the fewest scaffolds. Finally, 85 genomes were selected (last updated on 20 January 2026).

These sequences were introduced into AntiSMASH v8.0.4 [85], and the BGCs of the class II lanthipeptides were extracted from all the genomes. We chose the ‘relaxed’ option as the detection strictness parameter, and the ’Extra Features’ were set to all on. The protein sequences of the precursor peptide (LanA) and synthetase (LanM) were selected for subsequent analyses. BAGEL4 (http://bagel4.molgenrug.nl, accessed on 2 August 2026) [86] was used to validate some specific results. The web server Operon-mapper (https://biocomputo.ibt.unam.mx/operon_mapper/, accessed on 2 August 2026) [87] was used to identify operon organization within the BGC.

### 4.3. Analysis of Core Biosynthetic Genes of Class II Lanthipeptides

The LanA and LanM amino acid sequences were extracted from the AntiSMASH analysis. The precursor peptides and LanM enzymes were aligned using the MUSCLE platform (https://www.ebi.ac.uk/jdispatcher/msa/muscle, accessed on 2 August 2026) [88]. Subsequently, a conserved motif was localized in precursor peptides using the MEME suite [89], and that motif was selected based on the lower expectancy value. The LanM sequence logo was generated using the WebLogo 3 server (https://weblogo.threeplusone.com) [90].

The sequences of the mersacidin (MrsA: CAB60258.1; MrsM: CAB60261.1), haloduracin β (HalA2: WP_328017663.1; HalM2: WP_138090237.1), lichenicidin A2 (LicA2: P86720.1; LicM2: ADW08735.1), cinnamycin (CinA: CAD60520.1; CinM: CAD60521.1), cinnamycin B (CinA_B: WP_019631332.1; CinM_B: WP_019631331.1), kyamicin (KyaA: QDF63358.1; KyaM: QDF63359.1), duramycin (DurA: WP_071962206.1; DurM: WP_071962207.1), mathermycin (MatA: ARW80050.1; MatM: ARW80051.1), actagardine (GarA: ACR33052.1; GarM: ACR33053.1) and michiganin A (ClvA: CAN02023.1; ClvM: CAN02024.1) of both precursor peptides and the LanM enzyme were obtained from the NCBI database. The LanM and LanA sequences were aligned in MAFFTv7 [91] and the resulting sequences were concatenated with the LanA sequences in SeaView v5.1 [92].

Then, the resulting file was used to generate a phylogenetic tree with the maximum-likelihood (ML) method, using the Q.PFAM+F+I+R5 substitution model selected by ModelFinder v3.1.2 in IQ-Tree v3.1.2 [93,94]. A bootstrap analysis with 1000 resamples was conducted, and 1000 SH-aLRT replicates were used [95]. The interactive Tree Of Life (iTOLv7) platform [96] was used to visualize the results.

Due to the high identity percentage among several sequences, it was necessary to remove redundant sequences to obtain a more robust phylogenetic tree. Only genomic sequences with few scaffolds were selected for this purpose. However, all sequences were considered for subsequent analysis to examine differences in the distribution of other genes (which encoded tailoring enzymes and regulatory proteins) within their BGC.

Simultaneously, using the alignment of the concatenated LanA and LanM sequences, pairwise amino acid identity values were calculated using a custom Phyton script v3.13.5, excluding positions in the alignment that contained gaps. These results were visualized in a clustered heatmap using Seaborn library v0.13.2 with hierarchical clustering.

### 4.4. Genomic Cluster Family Analysis by BiG-SCAPE

The AntiSMASH v8.0.4 cluster analysis was downloaded, and the archives were imported into BiG-SCAPE v2.0.0 [97]. To select the best cutoff, 0.3, 0.5, and 0.8 were tested, and the commands ‘-include-singletons’ and ‘-mix’, were applied. The output archives were visualized and manually edited in Cytoscape v3.10.4. The phylogenetic tree for each analyzed family was directly obtained from the BiG-SCAPE v2.0.0 visualization.

To compare the BGC of Mersacidin, we used the BiG-SCAPE Query function to cutoffs of 0.3, 0.5, and 1.0. The query BGC (BGC0000527) was downloaded from MIBiG 4.0 [98]. Furthermore, a comparison was carried out between the set of analyzed BGCs and the MIBiG 4.0 repository.

### 4.5. Cluster Comparison

The BGCs from distinct groups were extracted from AntiSMASH v8.0.4 or BAGEL4 analysis in GBK or FASTA format, respectively. These files were introduced into the CAGECAT clinker (https://cagecat.bioinformatics.nl/tools/clinker, accessed on 2 August 2026) for cluster comparison [99]. Figures were edited manually. The multiple sequence alignments were performed in MUSCLE (https://www.ebi.ac.uk/jdispatcher/msa/muscle, accessed on 2 August 2026) and edited in Jalview v2.11.5.1 [100].

## 5. Conclusions

In the search for new compounds with potential applications across different fields, genome mining is a valuable strategy for prioritizing the expression of peptides with diverse characteristics. In this study, we conducted a systematic analysis of the actinopeptin family of lanthipeptides within the phylum Actinomycetota. This phylum is recognized as the most significant producer of this peptide category in genomic databases. However, we also observed heterogeneity in their possible biosynthetic pathways, which are shared by only a few genera. This diversity offers the opportunity to gain new insights into class II lanthipeptides in actinobacterial strains. Future studies will emphasize the description, characterization, and production of various BGCs within each group, with a particular focus on exploring another intriguing feature: the promiscuity of the LanM enzymes, as suggested by the presence of multicomponent clusters. We consider this a key study, not only for expanding our understanding of this fascinating group of lanthipeptides, but also as an opportunity to describe a new family of RiPPs.

## Figures and Tables

**Table 1:** Characteristics of the groups of the actinopeptin family. The main features of both the leader and core peptide are presented.

Group	Characteristics of the Leader Peptide	Number of Cysteine Residues in the Core Peptide	
I	Three amino acids separate the GG and XP[AV]GX motifs. Position −5 is an L Position −4 is a Y or F Position −3 can be V, I, or an uncharged amino acid	IA	Three
		IB	Four
II	Three or four amino acids separate the XP[AV]GX and Gx motifs. In this latter case, x can be a D, E, or S Position −6 is an aliphatic residue Position −5 is Y or F	The number ranges from four to five residues	
Multicomponent	The BGCs can produce more than one precursor peptide. In general, all peptides of the same system share similar characteristics with respect to their cleavage site.	The number ranges from four to five residues	
Unclassified	Variations in the cleavage site are present. Generally, not clustering in groups I or II, although the presence of motif Gx could be present. Metallopeptidases are encoded in some BGCs.	A maximum of four	

## Data Availability

Data will be made available on request.

## Supplementary Materials

The following supporting information can be downloaded at: https://www.mdpi.com/article/10.3390/antibiotics15080755/s1, Figure S1: Logo of the cyclization domain of the LanM enzyme; Figure S2: Heatmap of identity among sequences in the actinopeptins family. Figure S3: Gene Cluster Families (GCFs) containing sequences of the actinopeptins family; Figure S4: GNN of clusters within the phylum Actinomycetota; Table S1: Names and accession numbers of the genes from the phylum Actinomycetota obtained from the SNN analysis; Table S2: Gene Cluster Families identified in the BiG-SCAPE analysis; Table S3: Distribution of the different clusters and singletons from SNN analysis and GCFs in each group of the actinopeptins family stablished in this study; Table S4: Characteristics of the BGC analyzed in this study.

## Abbreviations

The following abbreviations are used in this manuscript:

RiPP	Ribosomally synthesized and post-translationally modified peptides
Lan	Lanthionine
MeLan	β-methyl-lanthionine
BGC	Biosynthetic gene cluster
DUF6229	Domain of unknown function 6229
SSN	Sequence similarity network
GNN	Genome neighborhood network
GCF	Gene cluster family
