# Supplementary material for: Diversity and Classification of the Actinopeptins: A New Family of Lanthipeptides Within the Genomes from the Phylum Actinomycetota
Source: Antibiotics (Basel). 2026 Aug 5;15(8):755. doi: 10.3390/antibiotics15080755 (PMC13509491; doi:10.3390/antibiotics15080755)
Supplement: Supplementary file 1 [file antibiotics-15-00755-s001.zip › antibiotics-4393075-supplementary.pdf]

Supplementary material

Supplementary Figure S1. Logo of the cyclization domain of the LanM enzyme. The red line shows the conserved motif CHG in the proteins. The Logo was generated from the curated alignment of LanM sequences.

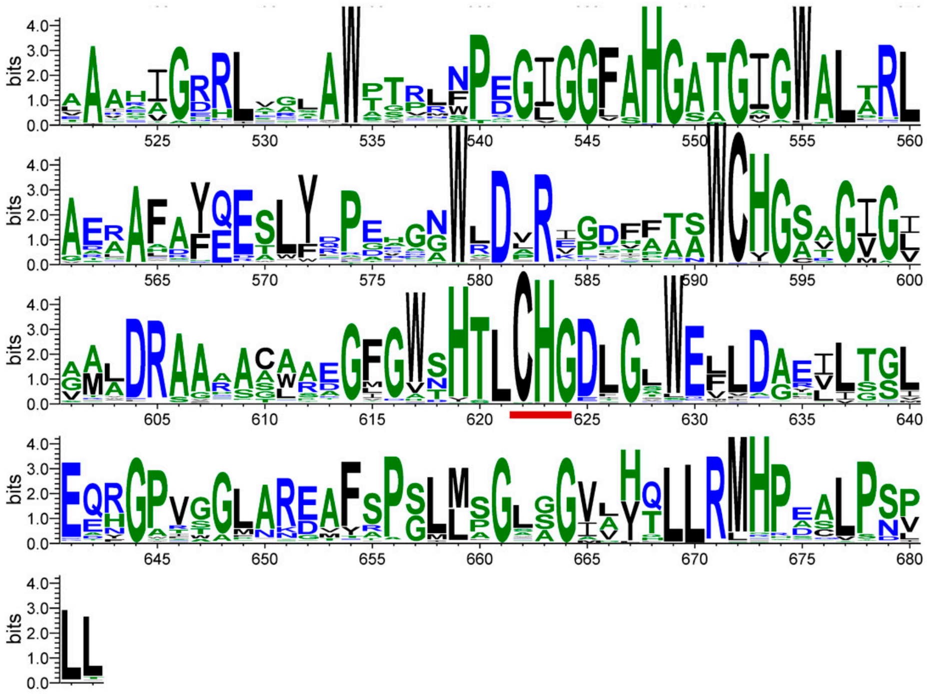

Supplementary Figure S2. Heatmap of identity among sequences in the actinopeptins family.

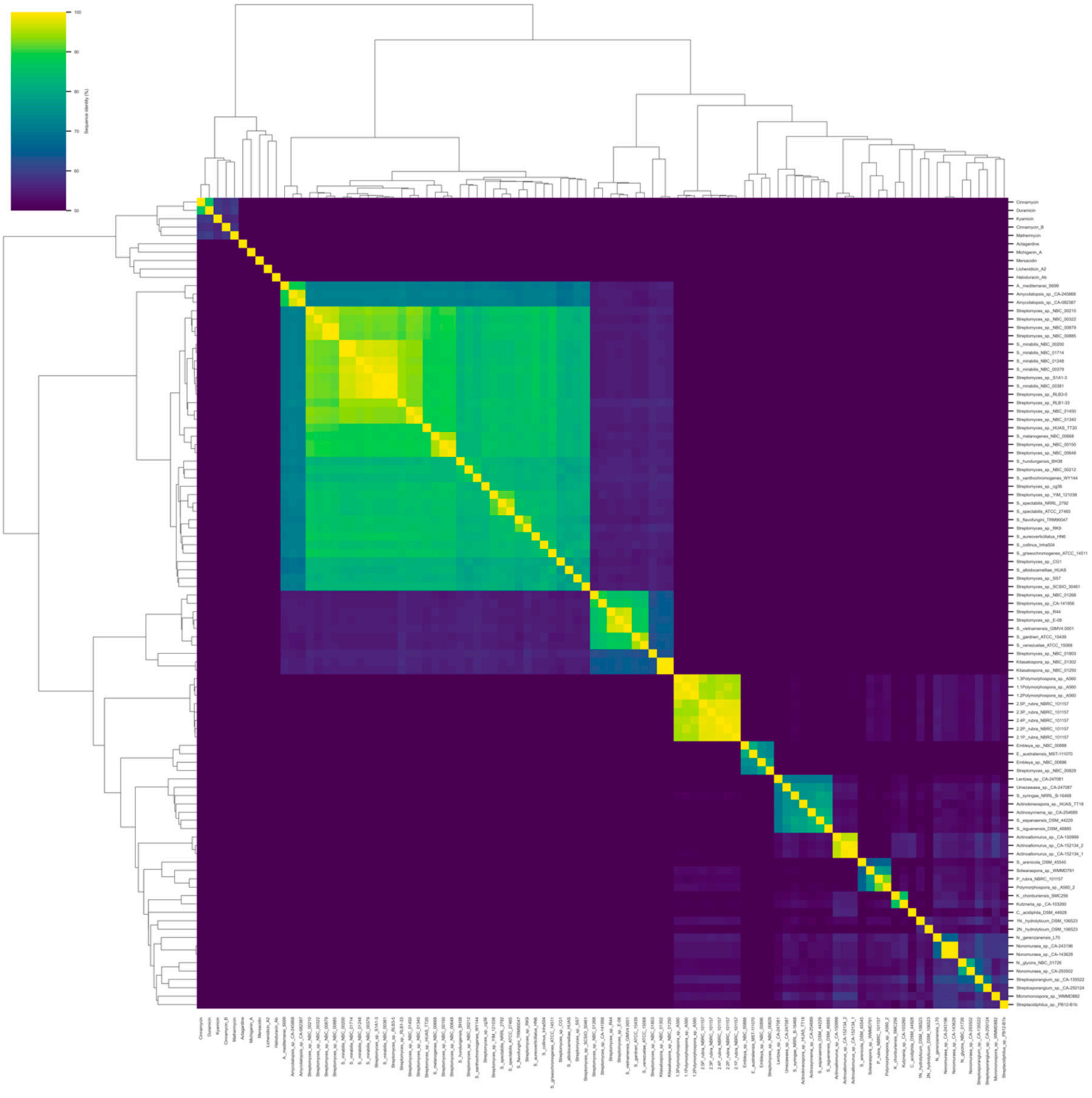

Supplementary Figure S3. Gene Cluster Families (GCFs) containing sequences of the actinopeptins family. Thirteen families were identified, each designated by a letter. The genera corresponding to each BGC are represented side to the GCF.

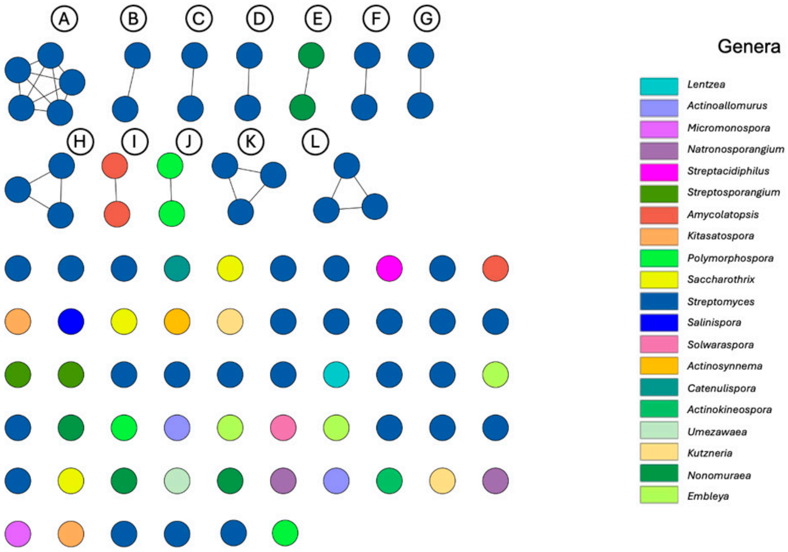

Supplementary Figure S4. GNN of clusters within the phylum Actinomycetota. The weight of each node represents the greater or lesser presence of this protein around the actinopeptin BGCs. Highlights the high abundance of several families in clusters 4, 5 and 6, and a low presence of proteins in cluster 1.

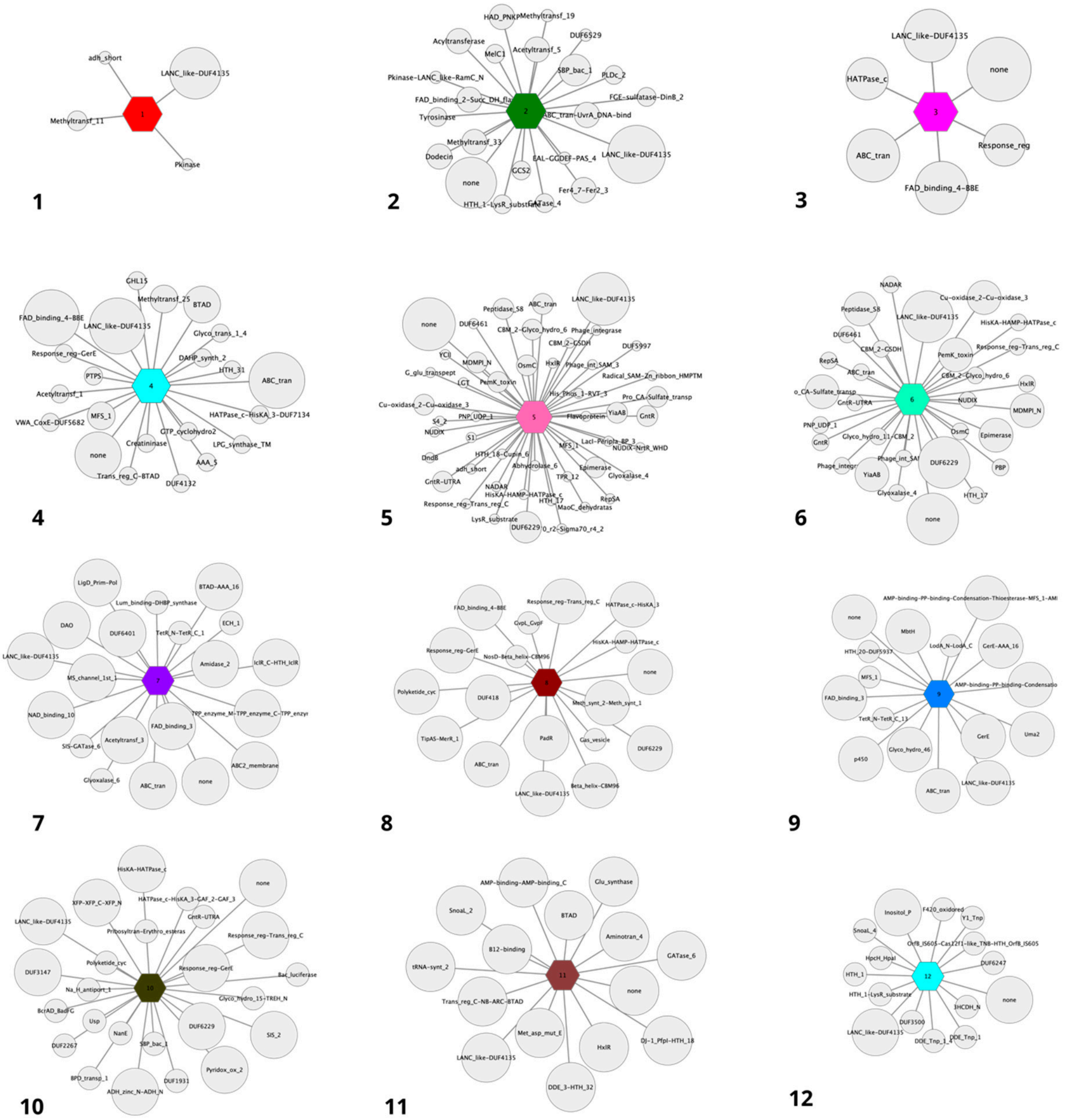

Supplementary Table S1. Names and accession numbers of the genes from the phylum Actinomycetota obtained from the SNN analysis.

Number of clusters	Actinobacteria	Accession number and sequences of the LanA in the UniProt database
1	*Streptomyces* sp. WM6378	>A0A0M9YC49<br>MPQTSVPASAVLNAWLSGAESAYGQLNPAGPLYVGGAAAEAALTDSSDSLYGFCSSPSGSHGSWCC
	Streptomyces sp. MMG1121	>A0A0N0SXX7<br>MPQSSVHAHAVLDAWLTGAESAFGQVNPAGPLYVGGAAAEAALTDPSDSLYGFCSSPSGSHGSWCC
	Actinobacteria bacterium OK006	>A0A0N1NGH3<br>MPQTSVATTAVLDAWLAGAESAYGQTNPAGPLYVGGAAAEAALTDPSDSLYGFCSSPSGSHGSWCCWSPAVTPGPFAEGL
	Streptomyces cellostaticus DSM 40189	>A0A101NQQ8<br>MSQPSAPTTAVLDAWLSGAESAYGRLNPAGPLYVGGTAAEAALTDPSDSLYGFCSSPSGSHGSWCC
	Streptomyces griseochromogenes DSM 40499	>A0A1B1AY60<br>MSQPSVPATAVLDAWLSGAESAYGRLNPAGPLYVGGRAAEAALTDPSDALYGFCSSPSGSHGSWCC
	Streptomyces sp. 2231.1	>A0A1H4QLF2<br>MSQNSVRTAAVLEAWLSGAETAYGEVNPAGPLYVGGTAAEAALTDPSDSLYGFCSSPSGSRGSWCC
	Streptomyces mirabilis	>A0A1I2K7A7<br>MPQTSVATTAVLDAWLAGAESAYGRTNPAGPLYVGGAAAEAALTDPSDSLYGFCSSPSGSHGSWCC
	Streptomyces sp. CB01883	>A0A1Q5KGP2<br>MRTAAVLEAWLSGAETAYGELNPAGPLYVGGTAAEAALTDPSDSLYGFCSSPSGSRGSWCC
	Streptomyces sp. IMTB 2501	>A0A1R0LEF2<br>MPQTRVSHTAVIEAWLSGAESAYGQSNPAGPLYVGGTAAEAALTDPSDSLYGFCSSPSGSHGSWCC

	Streptomyces alboflavus MDJK44	>A0A1Z1WS22<br>MHHTSVPTTAVLTAWLSGAESAYGQANPAGPLYVGGTAAEAALTDPSDSLYGFCSSPSGSHGSWCC
	Streptomyces diastatochromogenes DSM 40608	>A0A233S6N6<br>MPQTSVQTAAVLEAWLSGAESAFGQLNPAGPLYVGGAAAEAALTDPSDSLYGFCSSPSGSHGSWCC
	Streptomyces olivochromogenes NBRC 3561	>A0A250VW17<br>MPQISVATTAVLDAWLTGAESAYGQTNPAGPLYVGGAAAEAALTDPSDSLYGFCSSPSGSHGSWCC
	Streptomyces sp. Ag109_G2-15	>A0A286G6V5<br>MPQTSVQTAAVLEAWLSGAESAFGQLNPAGPLYVGGAAAEAALTDPSDSLYGFCSSPSGSHGSWCC
	Streptomyces sp. Ag82_O1-15	>A0A2A3JLA0<br>MPQTSVATTAVLDAWLAGAESAYGQTNPAGPLYVGGAAAEAALTDPSDSLYGFCSSPSGSHGSWCC
	Streptomyces sp. CB01201	>A0A2M9J4C0<br>MPQTSVPASTVLNAWLSGAESAYGQLNPAGPLYVGGAAAEAGLTDSSDSLYGFCSSPSGSHGSWCC
	Streptomyces sp. TLI_146	>A0A2N3W6U9<br>MPQTNVPATAVLNAWLSGAESAYGQSNPAGPLYVGGAAAEAALTDPTDSLYGFCSSPTGSRGSWCC
	Streptomyces sp. SDr-06	>A0A367HQ90<br>MPQTSVPASAVLDAWLSGAESACGQLNPAGPLYVGGAAAEAALTDPSDSLYGFCSSPSGSHGSWCC
	Streptomyces hundungensis BH38	>A0A387HDJ7<br>MSQNGVFASTVLHAWLSGAESAYGQLNPAGPLYVGGTAAEAALTDPSDSLYGFCSSPSGSRGSWCC
	Streptomyces sp. PanSC9	>A0A3N1IJW3<br>MSQNSVRTAAVLEAWLSGAETAYGELNPAGPLYVGGTAAEAALTDPSDSLYGFCSSPSGSRGSWCC
	Streptomyces sp. 1-11	>A0A510TFH4<br>MPQSSVHTAAVLEAWLSGAESAQGQINPAGPLYVGGAAAEAALTDPSDSLYGFCSSPSGSHGSWCC

	Streptomyces spectabilis	>A0A516RJL2<br>MPQTSVQSAVLEAWLSGAESAYGQANPAGPLYVGGTAAEAALTDPSDSLYGFCSSPSGSHGSWCC
	Streptomyces sp. BK340	>A0A559UBI2<br>MPQTSVQTAAVLEAWLSGAESAYGQLNPAGPLYVGGAAAEAALTDPSDSLYGFCSSPSGSHGSWCC
	Streptomyces sp. YIM 121038	>A0A5B7V6X7<br>MPQTSVQNAVLEAWLSGAESAYGQANPAGPLYVGGSAAEAALTDPSDSLYGFCSSPSGSHGSWCC
	Streptomyces spectabilis CECT 3146	>A0A5P2XI78<br>MPQTRVQSAVLEAWLSGAESAYGQANPAGPLYVGGSAAEAALTDPSDSLYGFCSSPSGSHGSWCC
	Streptomyces sp. RLB1-33	>A0A6H1KMI5<br>MPQTSVATAAVLDAWLAGAESAYGQTNPAGPLYVGGAAAEAALTDPSDSLYGFCSSPSGSHGSWCC
	Streptomyces sp. S1D4-11	>A0A6H1MY80<br>MPQTSVATTAVLDAWLAGAESAYGRTNPAGPLYVGGAAAEAALTDPSDSLYGFCSSPSGSHGSWCC
	Streptomyces echinatus CECT 3313	>A0A7W9UQH5<br>MSQSSVHTAAVLEAWLSGAESAYGQLNPAGPLYVGGAAAEAALTDPSDSLYGFCSSPSGSRGSWCC
	Streptomyces filipinensis JCM 4369	>A0A918MCT5<br>MPQTRVSHTAVIEAWLSGAESAYGQSNPAGPLYVGGTAAEAALTDPSDSLYGFCSSPSGSHGSWCC
	Streptomyces longispororuber JCM 4784	>A0A918ZPR5<br>MPQTRAHTDAVLEAWLSGAESAYGQTNPAGPLYVGGAAAEAALTDPSDSLYGYCSSPSGSHGSYCC
	Streptomyces alanosinicus JCM 4714	>A0A919D5R4<br>MPQSSVQAHAVLDAWLSGAESAFGQLNPAGPLYVGGSAAEAALTDPSDSLYGFCSSPSGSHGSWCC
	Streptomyces actinomycinicus RCU-197	>A0A937EHY7<br>MSQTSVHTTAVLEAWLSGAESAYGQLNPAGPLYVGGAAAEAALTDPSDSLYGFCSSPSGSRGSWCC

	Streptomyces sp. OK228	>A0AAJ4XV31<br>MPQTSVATTAVLDAWLAGAESAYGQTNPAGPLYVGGAAAEAALTDPSDSLYGFCSSPSGSHGSWCC
	Streptomyces sp. NBC_00212	>A0AAU1BIR5<br>MPQTSVPASAVLNAWLSGAESAYGQLNPAGPLYVGGAAAEAALTDSSDSLYGFCSSPSGSHGSWCC
	Streptomyces sp. NBC_00210	>A0AAU1CUS1<br>MPQTSVPTTAVLDAWLSGAESAYGQTNPAGPLYVGGAAAEAALTDPSDSLYGFCSSPSGSHGSWCC
	Streptomyces sp. NBC_00133	>A0AAU1PZP6<br>MPQTSVPTTAVLDAWLSGAESAYGQTNPAGPLYVGGAAAEAALTDPSDSLYGFCSSPSGSHGSWCC
	Streptomyces sp. NBC_00060	>A0AAU2GTG4<br>MPQTSVPATAVLNAWLSGAESAYGQSNPAGPLYVGGAAAEAALTDPSDALYGFCSSPSGSRGSWCC
	Streptomyces sp. NBC_00648	>A0AAU3AEL7<br>MPQTSVPATAVLNAWLSGAESAYGQSNPAGPLYVGGAAAEAALTDPSDALYGFCSSPSGSRGSWCC
	Streptomyces sp. NBC_00150	>A0AAU3SCU9<br>MPQTSVPATAVLNAWLSGAESAYGQSNPAGPLYVGGAAAEAALTDPSDALYGFCSSPSGSRGSWCC
	Streptomyces sp. NBC_00322	>A0AAU4KYZ4<br>MPQTSVPTTAVLDAWLAGAESAYGQTNPAGPLYVGGAAAEAALTDPSDSLYGFCSSPSGSHGSWCC
	Streptomyces sp. NBC_00271	>A0AAU4X032<br>MPQTSVATTAVLDAWLAGAESAYGQTNPAGPLYVGGAAAEAALTDPSDSLYGFCSSPSGSHGSWCC
	Streptomyces sp. NBC_00224	>A0AAU5FE63<br>MPQTSVPATAVLNAWLSGAESAYGQSNPAGPLYVGGAAAEAALTDPSDALYGFCSSPSGSRGSWCC
	Streptomyces sp. NBC_01450	>A0AAU6K4Q2<br>MPQTSVATTAVLDAWLAGAESAYGQTNPAGPLYVGGAAAEAALTDPSDSLYGFCSSPSGSHGSWCC
**2**	*Streptomyces vietnamensis*<br>GIMV4.0001	>A0A0B5IHJ9<br>MSIMSITRADEIVTAWLQGAETAFGADNPAGPLYVGGAAGEAALTDGTEALLTGCSSCTGSLHSYCC

	Streptomyces viridochromogenes	>A0A0L8J8Z8<br>MPVMSASRTDEIVTAWLRGAETAFGADNPAGPLYVGGAAGEAALTDATEALLTGCSSCTGSWHSYCC
	Streptomyces showdoensis ATCC 15227	>A0A2P2GU24<br>MSTTRTDDIVTAWLQGAETAFGADNPAGPLYVGGAAGEAALTDGTEALLTGCSSCTGSWHSYCC
	Streptomyces sp. PanSC19	>A0A3N1Q538<br>MPVMSATRTDEIVTAWLQGAETAFGADNPAGPLYVGGAAGEAALTDATEALLTGCSSCTGSWHSYCC
	Streptomyces sp. WAC01280	>A0A429RFT2<br>MPVMSATRTDEIVTAWLQGAETAFGAANPAGPLYVGGAAGEAALTDATEALLTGCSSCTGSWHSYCC
	Streptomyces gardneri NBRC 12865	>A0A4Y3RFX8<br>MPVISATRTDAIVTAWLQGAETAFGADNPAGPLYVGGAAGEAALTDATEALLTGCSSCTGSWHSYCC
	Streptomyces venezuelae	>A0A5P2AJW5<br>MPVISATRTDAIVTAWLQGAETAFGADNPAGPLYVGGAAGEAALTDATEALLTGCSSCTGSWHSYCC
	Streptomyces nymphaeiformis SFB5A	>A0A7W7U9F8<br>MSIMSITRADEIVTAWLQGAETAFGADNPAGPLYVGGAAGEAALTDGTEALLTGCSSCTGSLHSYCC
	Streptomyces sp. R44	>A0AB39SVF0<br>MSIMSTTRADEIVTAWLQGAETAFGADNPAGPLYVGGAAGEAALTDATEALLTGCSSCTGSLHSYCC
**3**	*Nonomuraea solani* CGMCC 4.7037	>A0A1H6DU76<br>MSCQVRSNGVYRPLVPATDHGAKFEKGASMTVTLERAEEIIAAWRTGDDPDSPVGPLLPGMYVESEITMTGPGGSGRCGTACSGSRTYQCC
	Nonomuraea sp. WAC 01424	>A0A429BGZ2<br>MTVTLERAEEIIAAWRAGDDPDSPAGPLMPGMYVESEITMTGPGGSGRCGTACSGSRTYQCC
	Nonomuraea endophytica DSM 45385	>A0A7W8EH48<br>MTVTLERAEEIILAWRTGEEPDSPVGPLLPGMYVESEITMTGPGHSGKCGTACSGSRTRYCC

	Nonomuraea muscovyensis DSM 45913<br><br>*Planomonospora venezuelensis* CECT 3303<br><br>*Nonomuraea* sp.	>A0A7X0C2S4<br>MTITLERAEEIIAAWRSGDEPDSPVGPLLPGMYVESEITMTGPGYSGKCGTACSGSYTRYCC<br><br>>A0A841D297<br>MTVTLERAEEIIAAWRSGDEADSPAGPVLPGMYVESEITMTGPGYSGRCGTACSGSRTYMCC<br><br>>A0A973SNM7<br>MTVTLERAEEIIAAWRAGDDPDSPVGPLMPGTYVESEITMTGPGGTGRCGTACSGSRTYMCC
**4**	*Saccharothrix carnea* CGMCC 4.7097<br><br>*Saccharothrix variisporea* DSM 43911<br><br>*Saccharothrix syringae* ATCC 51364<br><br>*Saccharothrix violaceirubra* DSM 45084<br><br>*Saccharothrix ecbatanensis* DSM 45486<br><br>*Saccharothrix espanaensis* ATCC 51144	>A0A2P8HZU7<br>MTTALLLEAEETVARWRAGLDEWDNPAGPLFANGEFAEGDIVACNPPGSRCSSCTASRPVYCC<br>>A0A495X991<br>MTTALLESAEELVARWRAGLDDVDNPAGPLFANGEFAEGDIVACNPPGSRCSSCTASRPVYCC<br>>A0A5Q0H495<br>MTAATLLDAEELVARWRAGLDELDNPAGPLFANGEFAEGDIVACNPPGSRCSSCTASAHVYCC<br>>A0A7W7T228<br>MTTALLPDAEELVARWRAGLDGIDNPAGPLFANGEFAEGDIVACNPPGSRCSACTASRPVYCC<br><br>>A0A7W9HI53<br>MTSAMLLDAEELVARWRAGLDEWDNPAGPLFANGEFAEGDIVACNPPGSRCSSCTASAPVYCC<br><br>>K0K3E8<br>MTAALLLDAEEMVARWRAGLDESDNPAGPLFANGEFAEGDIVACNPPGSRCSSCTASRPVYCC
	Microbispora cellulosiformans	>A0A5J5K8D3<br>MPLTLDRSEEIVSAWRSGADVEGWDSPAGPLFIGEYAESEITLTALDTRLASSCTCSRFCC

**5**	*Amycolatopsis echigonensis* NEAU-HEGS1-5<br><br>*Sphaerisporangium melleum* JCM 13064<br><br>*Sphaerisporangium rufum* NBRC 109079<br><br>*Streptosporangium carneum* VKM Ac-2007	>A0A5R8ZD47<br>MPLTLDRSEEIVSAWRSGADVEGWDSPAGPLFIGEYAESEITLTALDTRLASSCTCSRFCC<br><br>>A0A917R6E8<br>MPLTLDRSEEIVAAWRSGADVEGWDNPAGPLFIGDYAESEITLTALDTRMASSCTCSRFCC<br><br>>A0A919R0X7<br>MPVTLERSAEIVAAWRSGAEVEGWDSPAGPLFIGEYAESEITLTAMDTRLASSCTCSRFCC<br><br>>A0A9W6MAW0<br>MPVTLERSEEIVSAWRSGADVEGWDSPAGPLFIGDYAESEITLTALDTRLASSCTCSRFCC
**6**	*Microbispora cellulosiformans* Gxj-6<br><br>*Microbispora triticiradicis* NEAU-HEGS1-5<br><br>*Sphaerisporangium melleum* JCM 13064	>A0A5J5K7H9<br>MEGVRMPVTLDRSEEIVSAWRSGADVEGWDSPAGPLFIGEYAESEITLTALDTRRPSSCTGANTIPCC<br>>A0A5R8ZD57<br>MEGVRMPVTLDRSEEIVSAWRSGADVEGWDSPAGPLFIGEYAESEITLTALDTRRPSSCTGANTIPCC<br><br>>A0A917VJR3<br>MPVTLERSEEIVAAWRSGADVEGWDNPAGPLFIDEYAESEITLTAMDTQRPSSCTGARTIACC
**7**	*Nonomuraea gerenzanensis*<br><br>*Nonomuraea* sp. PA05	>A0A1M4EII3<br>MANLTAELAERIVAGWRGTAGPVEGWDSPAGPLFAGGDYAESDITMEAILGSAEAGAQPAAITVCGTVCTDSRPRVCC<br>>A0A5D0QHL7<br>MAPMTAELAERIVAGWRGDTRPVEGWDSPAGPLFAGGDYAESDITMEAILGSTEAGAQPAAVTVCGTVCTDSRPRVCC

**8**	*Polymorphospora rubra* NBRC 101157	>A0A810N9M8<br>MSTLAPERADEIIKSWLTGTDPVDGWDNPAGPLFLGGKYAEADITATGSGGGATDGITGCGWCTGSICNGFQVHCR
	Polymorphospora rubra NBRC 101157	>A0A810NDY7<br>MSTLAPERADEIIESWLTGTEPVDGWENPAGPLFLGGKYAEADITATGSGGSNSVTGCGWCSGSICNGMPIHCY
**9**	*Embleya scabrispora* NF3	>A0A1T3NP75<br>MRTASNATRNTVVDAWRSGAETTDGMENPAGSLYIGGSATEQGLTEIDVAAISCGTACSWSRTRACC
	Embleya hyalina NBRC 13850	>A0A401Z024<br>MRTASNATRNPVVDAWRSGAETADGVENPAGSLYIGGSATEQGLTEIDVAAISCGTACSWSRTRACC
**10**	*Sphaerisporangium siamense* DSM 45784	>A0A7W7D683<br>MPVTTERSEEIIAAWRSGADVDGWENPAGPLLSAYAESDLTTAFVETRRATACSGSFTHPCC
	Sphaerisporangium krabiense DSM 45790	>A0A7W8Z2S5<br>MPVTTERSEEIIAAWRSGAADIDGWENPAGPLLSRYAESELTTAFIETRRASACTGSFTHQCC
**11**	*Amycolatopsis mediterranei* U-32	>A0A0H3D871<br>MDSTAVLEGWLSGSESVHGLANPAGPLYVGGNAAEAALVDPTDMLYAFCSSPTGSHGSWCC
	Amycolatopsis mediterranei S699	>A0A9R0NZ72<br>MDSTAVLEGWLSGSESVHGLANPAGPLYVGGNAAEAALVDPTDMLYAFCSSPTGSHGSWCC
**12**	*Streptomyces* sp. HUCO-GS316	>A0A6G2INC7<br>MHTAAVLDAWLTGADTAYGLSNPAGPLYVGGAAAEAALTDPSDSLYGFCSSPTGSRGSWCC
	Streptomyces luteolifulvus HIT-DPA4	>A0A6H9UTW1<br>MHTAAVLDAWLTGADTAYGLSNPAGPLYVGGAAAEAALTDPSDSLYGFCSSPTGSRGSWCC

**Number of singletons**	**Actinobacteria**	**Access number of LanA sequence in the UniProt database**
**13**	*Micromonospora nigra* DSM 43818	>A0A1C6RCB4<br>MPTTLSVVDPVQAEEIITRWLTDTSVDGWENPAGDLFTGGEYALQEITMTISPPSSNCSSCTGSRPIACC
**14**	*Streptomyces pini* PL19	>A0A1I4HDC1<br>MPQATLTRADEIVAAWLDGAENSHGEPNPAGPLYVGGAATEAALVDATDALFTACSSCTGSLTTFCC
**15**	*Catenulispora* sp. 13_1_20CM_3_70_7	>A0A1Q7WCN9<br>MPQLEVLDRADQIIADWLASADGDSPAGPLFTGGQYAEAEIVGAAIIETRGTCSSCTASRGSYCC
**16**	*Kibdelosporangium aridum*	>A0A1W2AAM8<br>MTAAMLADAEELVARWREGLDLDSPAGPIFASGEFAEGDIVACNPPGSRCSSCTASRPVFCC
**17**	*Streptacidiphilus pinicola* MMS16-CNU450	>A0A2X0KEC8<br>MPSAAAAHPGRIVDAWLAGAETAFGKDNPAGPLFVGGGAAEAALTDSTDALMAFCSSPTGSYHSYCC
**18**	*Sphaerisporangium album* CCTCC AA 208026	>A0A367F9G1<br>MEHAEEIIAAWRRGVASAEGWENPAGPPLIGEYTEEEITMSLIETKRPSSCTAGGGFCC
**19**	*Embleya hyalina* NBRC 13850	>A0A401YMZ2<br>MRTTSDSSPVDEVVDAWRAGAVAPRGMRNPAGSLYIGGPATERGLTEIDVAAQTCGTVCSISRTRMCC
**20**	*Salinispora arenicola* NBRC *105043*	>A0A542XS59<br>MPPVASPHIDSSSAEEIINRWLSDTSLHGSDSPAGPVFTGGRYVVQEITATAVANWTYCSACTGSGPGTHCC
**21**	*Lentzea tibetensis* FXJ1.1311	>A0A563ESI1<br>MTTATLLKAEEIVAGWRSGLDFGDMMNPAGPMFASGEFAEGDIVACNPPGSRCSACTASRPVYCC

**22**	*Streptomyces* sp. SID3343	>A0A7K2Z029<br>MRTAAKDSAETVVNAWREGVETAAGAENPAGSLYIGGSATEAGLTDIDVAALTCGTACSGSRTRMCC
**23**	*Sphaerisporangium siamense*<br>DSM 45784	>A0A7W7D3M4<br>MPVTLERSEQIIAAWRSGADVDGWENPAGPLLSEYAESEITTAFGETRRPTACTGANTNPCC
**24**	*Sphaerisporangium krabiense*<br>DSM 45790	>A0A7W8Z2F7<br>MPVPLERSEQIIAAWRSGAADADGWENPAGPPLARYAESEITAAFAETRRPTACTGANTIPCC
**25**	*Polymorphospora rubra* NBRC<br>101157	>A0A810N764<br>MPTTTLPDLDRVPTQDLITRWLTDTPVDGWESPAGPLFTGGDYALQEITMTLGCPGSNCSSCTGSRPIQCC
**26**	*Polymorphospora rubra* NBRC<br>101157	>A0A810NA42<br>MSTLAPERTDEIIKSWLTGTELVDGWDSPAGPLFLGGRYVESDITATTTAGTVSGVIDGITW
**27**	*Polymorphospora rubra* NBRC<br>101157	>A0A810NDG5<br>MSTLAPERADEIIESWLTGTAPVDGWENPAGPLFNSGRYAEYDITATGGGGGITTHTGCGPCTGSYPIECY
**28**	*Polymorphospora rubra* NBRC<br>101157	>A0A810NFT3<br>MSTLAPERTDEIIKSWLTGIDPIDGWDNPAGPLFLGGKYAEADITATGGGGTGSISVTGCAWCTGSICNGRRILCA
**29**	*Allocatelliglobosispora scoriae*<br>DSM 45362	>A0A841BQF6<br>MSQEQAASEVAQWRSAAGKANPAGPLFSGGQYTEAELRMKTGRPTNRCGTACTWSATRYCC
**30**	*Natronosporangium hydrolyticum*<br>DSM 106523	>A0A895Y6M1<br>MSTLAPERTAAETVDAWRRGLDDSWENPAGPRFLGDYTESEITLTGGQTNDPATMGSLCTGGICTACC
**31**	*Natronosporangium hydrolyticum*<br>DSM 106523	>A0A895YCV9<br>MPTLTIEQVDEIIDGWRNGEEQDGLENPAGPLFSSSGYAESEITLTGTGYPGLSCGTGCSGSYDPATGMHYFCC

**32**	*Virgisporangium ochraceum* NBRC 16418	>A0A8J4A0Q0<br>MVMSDLTQEAADRIVASWRGSGPAVAEWDSPAGPVFAAGEFAESDITMEAIFAASGCPNPTPGTNLCGTACTYSAGRQCC
**33**	*Catenulispora* sp.	>A0A973P399<br>MKHVSKEKPAEKVHAWRNGTAADSPAGPLFTSGVYAESELTMTGAVLTAFCGTDCSYSHTRYCC
**34**	*Umezawaea endophytica* DSM 103496	>A0A9X2VP83<br>MTATALLEAEETVARWRAGLDDSPAGPLFASGEFAEGDIVACNPPGSRCSACTASRPVVCC
**35**	*Streptantibioticus silvisoli*	>A0AA90GZA7<br>MIKKEMDQVLAAWLSGSDSACGMENPAGPLFAGTVFSVTDSLITIEEPSITIEEPSVVGALITIEEPSITIEEPSLVGAL<br>ITIEEPSITIEEPSVVEALITIEEPSITIEEPSLVDSLITIEEPSITIEEPSLVGALITIEEPSITIEEPSLVGALITIE<br>EPSITIEEPSLVDSLITIEEPSITIEEPSLVEALITIEEPSITIEEPSLVDSLITIEEPSITIEEPSLVDMLITIEEPSI<br>TIEEPSLVDMFITIEEPSVLEALITIEEPSLVGALITIEEPSITIEEPGLVYA
**36**	*Kutzneria* sp. CA-103260	>A0AAT9LQR2<br>MSPTDLVTRWRTEPQTPDNPAGPLYVGEYAESELTSVIPPDTLHGCGTACTASRTRYCC
**37**	*Kitasatospora* sp. NBC_01250	>A0AAU5K6F1<br>MTMFTRSRTDALVAAWLSGAEGVDGLANPAGPLYVGGAAAEAALAESYEGILTHCSSCSASLHSYCC
**38**	*Salinispora arenicola* CNS-205	>A8M331<br>MPPVASPDIDISSAEEIINRWLSDTSLDGSDSPAGPLFTGGRYVVQEITATFGGTGTARCSSCTGSHRIGCC
**39**	*Catenulispora acidiphila* DSM 44928	>C7PW58<br>MSENEILDRTDAILSGWLGSTEDSPAGPLFTGGKYAEADIVGAVVILTRGTCSSCTASRGGECC
**40**	*Wenjunlia vitaminophila* ATCC 31673	>A0A0T6LQ97<br>MQAIPLAETKDIVSGWRAGAESVGAFDNPAGPLYVGGAATEAGLTRPDVAAVTGLSPCTGSGPIDCC
**41**	*Rhizocola hellebore* NBRC 109834	>A0A8J3QIQ2

		MKLLSPVQVDQVAASWRLGLDTAEDNPAGPLFLAGRYAVGEITASDPALNTQSQCQTAWLTNCPPMCHPIEDVGTAQ

Supplementary Table S2. Gene Cluster Families identified in the BiG-SCAPE analysis.

Family	Sequences	Accession number of the genomes	Localization of the BGC
A	*Streptomyces mirabilis* NBC_00381	GCF_036069355.1	1481496 – 1510571
	Streptomyces sp. S1A1-7	GCF_007113565.2	1184551 – 1213626
	Streptomyces sp. S1A1-3	GCF_007113465.1	796794 – 825867
	Streptomyces sp. RLB3-17	GCF_007113425.2	746812 – 775887
	Streptomyces sp. RLB3-5	GCF_007113445.1	636130 – 665205
B	*Streptomyces venezuelae* ATCC 15068	GCF_008642375.1	955170 – 978020
	Streptomyces gardneri ATCC 15439	GCF_015710995.1	7873974 - 7896824
C	*Streptomyces* sp. NBC_01340	GCF_035949105.2	9854357 – 9877210
	Streptomyces sp. NBC_01450	GCF_036227505.1	319720 – 342582
D	*Streptomyces sp.* NBC_00133	GCF_045160855.1	2093845 – 2154201
	Streptomyces sp. NBC_00210	GCF_045160975.1	2140914 – 2163749
E	*Nonomuraea* sp. CA-243196	CP191885	4617891 – 4640861
	Nonomuraea sp. CA-143628	NZ_CP192072.1	4601762 – 4667612
F	*Streptomyces spectabilis* NRRL 2792	GCF_007363395.1	9172472 – 9195346
	Streptomyces sp. YIM 121038	GCF_006088715.1	9765393 – 9788258
G	*Streptomyces* sp. NBC_00885	GCF_045207475.1	162508 – 185340
	Streptomyces sp. NBC_00879	GCF_045207445.1	106922 – 129757
H	*Streptomyces* sp. S1D4-23	GCF_007113485.2	1188613 – 1223995
	Streptomyces mirabilis NBC_01248	GCF_041435415.1	11012269 – 11047723
	Streptomyces mirabilis NBC_00379	GCF_036073155.1	10788259 – 10823725
I	*Amycolatopsis* sp. CA-082387	CP191952.1	10899742 – 10954541
	Amycolatopsis sp. CA-245868	CP191914.1	5398061 – 5452738
J (Multicomponent system)	*Polymorphospora* sp. A560	GCF_045781765.1	5013598 – 5036478
	Polymorphospora rubra NBRC 101157	GCF_039530265.1	7968142 – 7991022
K	*Streptomyces sp.* NBC_00271	GCF_036178845.1	1010128 – 1040485
	Streptomyces mirabilis NBC_01714	GCF_036248315.1	1341967 – 1372324
	Streptomyces mirabilis NBC_00200	GCF_036202585.1	1031882 – 1062239
L	*Streptomyces* sp. NBC_00648	GCF_045207255.1	434259 – 457103
	Streptomyces sp. NBC_00150	GCF_045206275.1	8188459 – 8211303
	Streptomyces sp. NBC_00224	GCF_041435195.1	8188461 – 8211305

Supplementary Table S3. Distribution of the different clusters and singletons from SNN analysis and GCFs in each group of the actinopeptins family stablished in this study.

<table>
<tr><th colspan="2" rowspan="3">Group</th><th colspan="3">Analyses</th></tr>
<tr><th colspan="2">SSN[1]</th><th rowspan="2">GCF[2]</th></tr>
<tr><th>Clusters</th><th>Singletons</th></tr>
<tr><td rowspan="2">I</td><td>IA</td><td>2, 7, 9</td><td>13, 14, 15, 19, 20, 22, 29, 36, 38, 39</td><td>B</td></tr>
<tr><td>IB</td><td>1, 11, 12</td><td>17, 40</td><td>A, C, D, F, G, H, I, K, L, M</td></tr>
<tr><td colspan="2">II</td><td>3, 4, 5, 6</td><td>16, 18, 21, 34, 36</td><td>E</td></tr>
<tr><td colspan="2">Multicomponent</td><td>8</td><td>25, 26, 27, 28, 30, 31</td><td>J</td></tr>
<tr><td colspan="2">Unclassified</td><td>10</td><td>23, 24, 32, 33, 35, 41</td><td>---</td></tr>
</table>

[1]**SSN:** Sequence similarity network. The numbers correspond to different clusters or singletons identified in the EFI-EST analysis (see Figure 1 and Supplementary Table 1).

[2]**GCF**: Gene cluster families. Each label corresponds to different families localized in the BiG-SCAPE analysis (see Supplementary Figure S2 and Supplementary Table 2).

Supplementary Table S4. Characteristics of the BGC analyzed in this study. The data were obtained from an analysis performed using AntiSMASHv8.0. Each BGC has been grouped according to the classification presented in this study. Unclassified sequences are shown in gray. NS: Not specified source.

Group	Actinobacteria/Accession number	Number of ORF	Hybrid	Family of regulatory protein	Tailoring enzymes, transporter proteins and other related sequences	Isolation source
**IA**	*Embleya* sp. NBC 00896<br>(GCF_045207785.1)	17	No	1 TetR<br>1 MarR<br>CPR regulator protein	1 Aldo/keto reductase family oxidoreductase<br>1 MMPL domain-containing transport protein<br>1 ABC transporter protein	Soil
	Embleya sp. NBC 00888<br>(GCF_045207655.1)	9	NRPS	1 LuxR	1 Drug resistance transporter<br>1 PCMT (carboxymethyl transferase)<br>1 Class A 1-beta lactamase	NS
	E. australiensis MST-111070<br>(GCF_051903075.1)	14	NRPS	1 LuxR	1 Glycosyl hydrolase family 46<br>1 ABC transporter protein	Soil
	Streptomyces sp. NBC_00829<br>(GCF_045161795.1)	24	NRPS	--	1 WD-40 repeat-containing protein<br>1 ABC transporter protein	NS
	Streptomyces sp. NBC_01803<br>(GCA_035917415.1)	25	Butyrolactone	1MarR	1 Drug resistance transporter (EmrB/QacA)<br>1 Acetyl-CoA carboxylase	Soil
	Kitasatospora sp. NBC_01250<br>(GCF_036226465.1)	28	RiPP-containing azole	1 PadR<br>2 TetR	1 Drug resistance transporter (EmrB/QacA)<br>1 DAO (D-amino acid oxidase)<br>1 PCMT (Protein L-isoaspartate(D-aspartate) O-methyltransferase)	NS
	Kitasatospora sp. NBC_01302<br>(GCF_035976515.1)	26	RiPP-containing azole<br>Class I lanthipepide	1 PadR<br>2 TetR	1 Drug resistance transporter (EmrB/QacA)<br>1 DAO (D-amino acid oxidase)<br>1 PCMT (Protein L-isoaspartate(D-aspartate) O-methyltransferase)<br>1 Class A Beta-lactamase	NS

	Streptomyces sp. CA-141956 (CP191948.1)	20	No	1 Response regulator<br>1 Helix-turn-helix domain protein	1 Aldo/keto reductase family oxidoreductase<br>1 Sodium: dicarboxylate symporter family<br>1 Tyrosine phosphatase<br>2 Transposases (IS481)<br>1 Phosphate acetyl/butaryl transferase	Soil
	Streptomyces sp. NBC_01268 (GCF_036240795.1)	27	No	1 TetR<br>1 LysR	1 Beta-lactamase protein<br>1 Short-chain dehydrogenase/reductase (SDR)<br>1 NAD-dependent epimerase/dehydratase	NS
	Streptomyces venezuelae ATCC 15068 (GCF_008642375.1)	25	Melanin	1 LysR	1 Acyltransferase<br>1 Sugar-binding lipoprotein<br>1 ABC transporter protein<br>1 DAO (D-amino acid oxidase)	Soil
	Streptomyces gardneri ATCC 15439 (GCF_015710995.1)	23	Melanin	1 LysR	1 ABC transporter protein<br>1 DAO (D-amino acid oxidase)<br>1 Sugar-binding lipoprotein<br>1 Acyltransferase<br>1 Ergothioneine biosynthesis protein EgtB	Soil
	Streptomyces sp. E-08 (GCF_049913095.1)	21	No	--	1 Peptidase S8 alkaline serine protease (subtilase family)	Maize endosphere
	Streptomyces vietnamensis GIMV4.0001 (GCF_000830005.1)	23	Class IV lanthipeptide<br>Terpene	---	1 Putative transposase of IS4/5 family (DUF4096)	Soil
	Streptomyces sp. R44 (GCF_041053105.1)	18	No	---	1 Alkaline serine protease (subtilase family)<br>1 Peptidase_S9	Rhizosphere of tomato
	Nonomuraea gerenzanensis L70 (GCF_020215645.1)	20	No	1 TetR<br>1 SARP<br>1 IclR<br>1 NmrA	1 ABC transporter<br>1 GCN5-related N-acetyltransferase<br>1 Monooxygenase FAD-binding<br>1 Pyruvate oxidase/decarboxylase<br>1 FAD-dependent oxidoreductase	Soil

	Micromonospora sp. WMMD882 (GCF_027497255.1)	17	NRPS	1 GntR	1 Aldehyde dehydrogenase<br>Fe-ADH<br>1 Haloalkane dehalogenase<br>3 ABC transporters<br>1 Nuclear transport protein factor 2<br>1 FAD-dependent monooxygenase	From *Trididemnum orbiculatum*
	Catenulispora acidiphila DSM 44928 (GCF_000024025.1)	15	No	1 TetR<br>1 LacI	1 Enoyl-CoA hydratase<br>1 Acetyl-CoA carboxylase<br>1 Biotin carboxylase<br>1 ABC transporter	Forest soil
	Salinispora arenicola DSM 45545 (GCF_000384275.1)	29	No	1 RNA polymerase, sigma-24 subunit (ECF subfamily)<br>1 SARP<br>1 GntR<br>1 regulator XRE-domain HTH	1 Aminotransferase	Sea
**IB**	*Amycolatopsis mediterranei* S699 (GCF_000220945.1)	18	NRPS	1 SARP	1 Transposase<br>1 AMP-dependent synthetase and ligase	Soil
	Amycolatopsis sp. CA-082387 (CP191952.1)	13	NRPS	---	1 Ornithine cyclodeaminase/mu-crystallin family protein<br>1 2-Oxoglutarate-Fe(II) oxygenase superfamily protein<br>1 Thioesterase domain	Soil from forest
	Amycolatopsis sp. CA-245868 (CP191914.1)	20	NRPS	1 SARP	1 Amidohydrolase family protein<br>1 Ornithine cyclodeaminase/mu-crystallin family protein<br>1 2-Oxoglutarate-Fe(II) oxygenase superfamily protein<br>1 Thioesterase domain	Soil from forest
	Streptomyces sp. NBC_00212 (GCF_045206365.1)	26	Terpene	---	---	Soil

	Streptomyces sp. SS7 (GCF_037993025.1)	27	Type 3 PKS	2 TetR	1 Phenylalanine-specific permease<br>1 GCN5-related N-acetyltransferase<br>2 Major facilitator transporters	From *Crinum latifolium*
	Streptmyces hundungensis BH38 (GCF_003627815.1)	23	No	1 LuxR<br>1 TetR<br>1 LysR	1 Polysaccharide deacetylase<br>1 Isochorismatase<br>1 Oxidoreductase dependent of NAD(P)/FAD	Soil
	Streptomyces sp. CG1 (GCF_041080625.1)	28	No	2 IclR<br>1 AsnC	1 Aldehyde oxidase and xanthine dehydrogenase<br>1 Crotonyl-CoA reductase / alcohol dehydrogenase<br>1 Glycerol kinase<br>1 Monooxygenase FAD-binding<br>1 PP2C family protein-serine/threonine phosphatase	Soil
	Streptomyces sp. cg36 (GCF_041080675.1)	27	No	1 MarR<br>1 RNA polymerase, sigma-24 subunit (ECF subfamily)<br>1 MerR	1 Homoserine/threonine efflux protein<br>1 NAD-dependent epimerase/dehydratase	Soil
	Streptomyces sp. YIM 121038 (GCF_006088715.1)	23	No	1 Sensor histidine kinase<br>1 LuxR family DNA-binding response regulator<br>1 TetR family transcriptional regulator<br>1 HxlR-like helix-turn-helix protein	1 Erythromycin esterase<br>1 MgtC family protein (virulence factor)<br>1 Permease for cytosine/purines, uracil, thiamine, allantoin<br>1 Amidohydrolase family protein<br>1 NUDIX domain containing protein<br>1 Alpha/beta hydrolase domain-containing protein<br>1 Alkaline phosphatase<br>2 MT0933-like antitoxin protein<br>1 Beta-lactamase	Soil
	Streptomyces spectabilis ATCC 27465 (GCF_008704795.1)	23	No	1 SARP<br>2 Unknow family (SMCOG1167)1 TetR	1 O-methyltransferase<br>1 Alfa/beta hydrolase protein	Soil
	Streptomyces spectabilis NRRL 2792 (GCF_007363395.1)	27	No	1 Sensor histidine kinase	1 Amidohydrolase	Soil

				1 LuxR family DNA-binding response regulator<br>1 TetR<br>2 Unknown family regulators	1 Alpha/beta hydrolase domain-containing protein<br>1 NmrA family protein	
	Streptomyces collinus Inha504 (GCF_032927875.1)	25	No	2 TetR	1 ABC transporter protein<br>1 DAO (D-amino acid oxidase)<br>1 Aldehyde dehydrogenase<br>1 Serine/threonine protein kinase<br>1 Short-chain dehydrogenase/reductase SDR<br>1 PP2C family protein-serine/threonine phosphatase	Soil
	Streptomyces griseochromogenes ATCC 14511 (GCF_001542625.1)	23	Type 1 PKS<br>NRPS<br>NAPAA	1 MarR	1 ABC transporter protein<br>2 Short-chain dehydrogenase/reductase SDR<br>1 APH protein<br>1 Aldehyde dehydrogenase<br>1 ABC permease system	NS
	Streptomyces albidocamelliae HUAS 14-6 (GCF_025642175.1)	19	No	2 TetR<br>1 MarR<br>1 LysR	2 Short-chain dehydrogenase/reductase SDR<br>1 Serine/threonine protein kinase<br>1 HAD-superfamily hydrolase<br>1 GCN5-related N-acetyltransferase	Soil
	Streptomyces aureoverticillatus HN6 (GCF_010669165.1)	22	No	1 RNA polymerase, sigma-24 subunit (ECF subfamily)<br>1 Hydrogen peroxide sensitive repressor	1 Carboxylate-amine ligase	Soil
	Streptomyces sp. RK9 (GCF_045070545.1)	17	Terpene<br>NRPS<br>Class III lanthipeptide	1 Hydrogen peroxide sensitive repressor	1 Aminotransferase<br>1 NAD-dependent epimerase/dehydratase<br>1 Arginosuccinate lyase/adenyl succinate lyase<br>1 methyltransferase	Soil

**IB**	*Streptomyces flavofungini* TRM90047 (CP128846.1)	16	NRPS<br>Terpene<br>Class III lanthipeptide	---	1 Aminotransferase<br>1 NAD-dependent epimerase/dehydratase<br>2 Arginosuccinate lyase/adenyl succinate lyase<br>1 Methyltransferase	Pear garden topsoil
	Streptomyces xanthochromogenes WY144 (GCF_036541015.1)	20	No	---	1 Aldo/keto reductase family oxidoreductase<br>1 Aldehyde dehydrogenase<br>1 PP2C family protein-serine/threonine phosphatase	Soil
	Streptomyces sp. SCSIO 30461 (GCF_037023745.1)	18	No	---	1 Iron compound ABC transporter<br>1 Glucose-1-phosphate adenylyl/thymidylyltransferase	Sediment
	Streptomyces sp. NBC_00322 (GCF_036147535.1)	20	No	1 Sensor histidine kinase<br>1 Response regulator	1 ABC transporter protein<br>1 WD-40 repeat-containing protein	Soil
	Streptomyces sp. NBC_00210 (GCF_045160975.1)	19	No	---	1 Glucose-1-phosphate adenylyl/thymidylyltransferase<br>1 NAD-dependent epimerase/dehydratase<br>1 Binding-protein-dependent transport systems<br>1 Flavodoxin	NS
	Streptomyces sp. NBC_00885 (GCF_045207475.1)	23	No	1 AraC<br>1 MerR<br>1 TetR	1 Pyruvate oxidase/decarboxylase<br>1 3-oxoacyl-(acyl carrier protein) synthase III<br>1 AB hydrolase<br>1 FAD-binding oxidoreductase	Soil
	Streptomyces sp. NBC_00879 (GCF_045207445.1)	25	No	1 AraC<br>1 MerR<br>1TetR	1 pyruvate oxidase/decarboxylase<br>1 3-oxoacyl-(acyl carrier protein) synthase III<br>1 FAD-binding oxidoreductase	Soil
	Streptomyces sp. NBC_00648 (GCF_045207255.1)	31	No	1 RNA polymerase, sigma-24 subunit (ECF subfamily)<br>1 AraC<br>1 TetR	2 Major facilitator transporters<br>1 Homoserine/threonine efflux protein<br>1 Monooxygenase FAD-binding<br>1 Methyltransferase<br>1 NmrA family protein<br>1 NAD-dependent epimerase/dehydratase	Soil

	Streptomyces melanogenes<br>NBC_00668<br>(GCF_036226805.1)	25	No	1 TetR<br>1 AraC	1 NAD-dependent epimerase/dehydratase<br>2 Major facilitator transporters<br>1 NmrA family protein<br>1 Methyltransferase<br>1 Monooxygenase FAD-binding (DAO)<br>1 Homoserine/threonine efflux protein	NS
	Streptomyces sp. NBC_00150<br>(GCF_045206275.1)	29	No	1 TetR<br>1 AraC<br>1 RNA polymerase, sigma-24 subunit (ECF subfamily)	1 NAD-dependent epimerase/dehydratase<br>2 Major facilitator transporters<br>1 NmrA family protein<br>1 Methyltransferase<br>1 Monooxygenase FAD-binding<br>1 Homoserine/threonine efflux protein	Soil
	Streptomyces sp. NBC_01340<br>(GCF_035949105.2)	28	No	1 LacI	1 GCN5-related N-acetyltransferase	NS
	Streptomyces sp. NBC_01450<br>(GCF_036227505.1)	24	No	1 AraC<br>1 LacI	1 Acyl-CoA dehydrogenase<br>1 L-carnitine dehydratase/bile acid-inducible<br>1 Enoyl-CoA hydratase<br>1 3-hydroxybutyryl-CoA dehydrogenase<br>1 AMP-dependent synthetase and ligase<br>1 Sugar transport protein<br>1 ABC transporter protein	NS
	Streptomyces mirabilis<br>NBC_00379<br>(GCF_036073155.1)	21	Terpene	1 LacI	1 Oxidoreductase<br>1 Sugar transport protein<br>1 ABC transporter, carbohydrate uptake<br>1 PP2C family protein-serine/threonine phosphatase	Soil
	Streptomyces sp. RLB1-33<br>(GCA_012273535.2)	16	Terpene	1 LacI	3 Transposases<br>1 ABC transporter protein<br>1 Sugar transport protein<br>1 Oxidoreductase (GFO/IDH/MocA)<br>1 PP2C family protein-serine/threonine phosphatase	Microscale soil grain

	Streptomyces mirabilis NBC_01714 (GCF_036248315.1)	21	Terpene	---	1 Alpha beta hydrolase<br>1 Methyltransferase<br>1 Protein phosphatase 2C	NS
	Streptomyces mirabilis NBC_00200 (GCF_036202585.1)	21	Terpene	---	2 Transposases<br>1 Alpha/beta hidrolasa<br>1 Methyltransferase	NS
	Streptomyces sp. RLB3-5 (GCF_007113445.1)	24	Terpene	---	1 Bacterial alpha-L-rhamnosidase 6 hairpin glycosidase domain<br>1 Glycosyl hydrolase family 76<br>1 RibD C-terminal domain<br>1 Stage II sporulation protein E (SpoIIE)<br>1 Oxidoreductase family, C-terminal alpha/beta domain	Microscale soil grain
	Streptomyces sp. S1A1-3 (GCF_007113465.1)	23	Terpene	---	1 Bacterial alpha-L-rhamnosidase 6 hairpin glycosidase domain<br>1 Glycosyl hydrolase family 76<br>1 RibD C-terminal domain<br>1 Stage II sporulation protein E (SpoIIE)<br>1 Oxidoreductase family, C-terminal alpha/beta domain<br>1 Glyceraldehyde 3-phosphate dehydrogenase, NAD binding domain<br>1 Cyclic nucleotide-binding domain- Phage capsid-like protein	Microscale soil grain
	Streptomyces mirabilis NBC_01248 (GCF_041435415.1)	23	Terpene	1 LacI	1 Oxidoreductase (GFO/IDH/MocA)<br>1 sugar transport protein<br>1 ABC transporter, carbohydrate uptake<br>1 transporter, carbohydrate uptake<br>1 PP2C family protein-serine/threonine phosphatase	NS
	Streptomyces mirabilis NBC_00381 (GCF_036069355.1)	23	Terpene	---	1 Oxidoreductase (GFO/IDH/MocA)<br>1 Serine/threonine-protein phosphatase	Soil

	Streptomyces sp. NBC_00224 (GCF_041435195.1)	29	No	1 TetR<br>1 AraC<br>1 RNA polymerase, sigma-24 subunit (ECF subfamily)	1 NAD-dependent epimerase/dehydratase<br>2 Major facilitator transporters<br>1 NmrA family protein<br>1 Methyltransferase<br>1 FAD-binding monooxygenase	Soil
	Streptomyces sp. NBC_00271 (GCF_036178845.1)	22	Terpene	---	1 Transposase<br>1 Alpha-beta hydrolase<br>1 Methyltransferase	Soil
	Streptomyces sp. NBC_01340 (GCF_035949105.2)	28	No	1 LacI	1 GCN5-related N-acetyltransferase<br>1 Oxidoreductase (GFO/IDH/MocA)	NS
	Streptomyces sp. NBC_00133 (GCF_045160855.1)	21	NRPS	---	1 Major facilitator transporter<br>1 Aminotransferase<br>1 Binding-protein-dependent transport systems<br>1 Flavodoxin	NS
	Streptomyces qianjiangensis HUAS TT20 (GCA_025642155.1)	29	No	1 HxlR-like helix-turn-helix<br>1 RNA polymerase, sigma-24 subunit (ECF subfamily)<br>1 Sensor histidine kinase<br>1 LuxR family DNA-binding response regulator	1 Histidine phosphatase superfamily protein<br>1 Flavodoxin-like fold contain protein<br>1 DUF4177-containing protein<br>1 DUF6629-containing protein<br>1 Glycosyl transferase family 2<br>1 DUF6215<br>1 Restriction endonuclease<br>1 S-adenosyl methyltransferase<br>1 Type II CAAX prenyl endopeptidase Rce1-like<br>1 S1 RNA binding domain	Rhizosphere soil
**IB**	*Streptomyces* sp. RLB3-17 (GCF_007113425.2)	22	Terpene	---	1 Bacterial alpha-L-rhamnosidase 6 hairpin glycosidase domain protein<br>1 Glycosyl hydrolase family 76<br>1 RibD C-terminal domain protein<br>1 Stage II sporulation protein E (SpoIIE)<br>1 Oxidoreductase (GFO/IDH/MocA)<br>1 Glyceraldehyde 3-phosphate dehydrogenase	Microscale soil grain
	Streptomyces sp. S1D4-23 (GCF_007113485.2)	21	Terpene	1 LacI	1 Bacterial alpha-L-rhamnosidase 6 hairpin glycosidase domain protein	Microscale grain soil

					1 Glycosyl hydrolase family 76<br>1 RibD C-terminal domain<br>1 Sodium/hydrogen exchanger family protein<br>1 Stage II sporulation protein E (SpoIIE)<br>1 Periplasmic binding protein domain<br>2 Xylose isomerase-like TIM barrel protein<br>2 Sugar transporters<br>1 Oxidoreductase (NAD-binding Rossmann fold)<br>1 Glyceraldehyde 3-phosphate dehydrogenase	
	Streptomyces sp. S1A1-7<br>(GCF_007113565.2)	24	Terpene	---	1 Bacterial alpha-L-rhamnosidase 6 hairpin glycosidase domain protein<br>1 Glycosyl hydrolase family 76<br>1 RibD C-terminal domain protein<br>1 Sodium/hydrogen exchanger family protein<br>1 Stage II sporulation protein E (SpoIIE)<br>1 Oxidoreductase protein<br>1 Glyceraldehyde 3-phosphate dehydrogenase	Microscale grain soil
**Multicomponent**	*Natronosporangium hydrolyticum* DSM 106523<br>(GCF_016925615.1)	23	No	1 AsrR<br>1 Hydrogen peroxide sensitive repressor<br>1 SARP<br>1 LuxR	3 ABC transporters<br>1 FAD linked oxidase domain protein<br>1 Acyl-CoA dehydrogenase<br>1 Helix-turn-helix domain protein	Soda solonchak soil
		22	No	1 Sensor histidine kinase<br>1 Response regulator	2 Methyltransferases<br>1 FAD linked oxidase domain protein	
	Polymorphospora sp. A560<br>(GCF_045781765.1)	21<br>3 LanA	No	1 Sensor histidine kinase<br>1 Response regulator<br>1 DUF418<br>1 Transcriptional regulator PadR-like family	1 FAD linked oxidase domain protein<br>1 ABC<br>1 AI-2E family transporter<br>1 Transposase	Soil

		19	NRPS	2 tRNA-splicing ligase RtcB<br>1 SARP<br>1 ArsR<br>1 Activator of Hsp90 ATPase homolog 1-like protein<br>1 LacI	1 Penicillin amidase<br>2 ABC<br>2 Binding-protein-dependent transport system inner membrane component<br>1 Extracellular solute-binding protein family 5	
	Polymorphospora rubra NBRC 101157 (GCF_039530265.1)	24<br>5 LanA	No	1 Sensor histidine kinase<br>1 LuxR response regulator<br>1 Response regulator<br>1 PadR-like regulator<br>1 MerR	1 DUF418<br>1 FAD linked oxidase domain protein<br>1 ABC transporter<br>1 S-methyltransferase<br>1 Polyketide cyclase / dehydrase and lipid transporter	Soil surrounding mangrove roots
		17	No	1 LacI<br>1 SARP<br>1 AraC	1 2-hydroxyacid dehydrogenase<br>1 FAD linked oxidase domain protein<br>1 ABC transporter	
	Actinoallomurus sp. CA-152134 (CP191934.1)	27<br>2 LanA	No	1 GntR<br>1 AraC<br>1 Helix-turn-helix domain protein<br>1 Histidine kinase-like protein<br>1 MerR<br>1 Penicillinase repressor	1 AB hydrolase protein<br>3 transposases<br>1 DUF5753<br>1 Sulfotransferase<br>1 peptidase family M48/M56	Soil
**II**	*Nonomuraea* sp. CA-243196 (CP191885)	22	No	1 IclR<br>1 LuxR<br>1 LysR	1 NmrA family protein<br>1 DUF6807<br>1 Oxidoreductase family protein<br>1 Monooxygenase FAD-binding<br>1 Dioxygenase	Soil from a waterlogged area

					1 Argininosuccinate lyase/adenylosuccinate lyase<br>1 Alpha/beta hydrolase family<br>1 ABC transporter<br>1 Glu/Leu/Phe/Val dehydrogenase<br>1 Putative transposase<br>1 DUF4242<br>1 S-adenosyl methyltransferase	
	Nonomuraea sp. CA-143628 (NZ_CP192072.1)	21	NRPS	1 IclR transcriptional regulator<br>1 SARP<br>1 LysR	1 NmrA-like family protein<br>1 DUF6807<br>1 Oxidoreductase family<br>1 Monooxygenase FAD-binding<br>1 Dioxygenase<br>1 Arginosuccinate lyase/adenyl succinate lyase<br>1 alpha/beta hydrolase family<br>1 ABC transporter<br>1 Glu/Leu/Phe/Val dehydrogenase<br>1 Putative transposase<br>1 S-adenosyl methyltransferase	Soil
	Streptacidiphilus sp. PB12-B1b (GCF_014084125.1)	27	No	4 TetR<br>1 Unknown family regulator transcriptional	1 Methyltransferase<br>1 Major facilitator transporter<br>1 Iron compound ABC transporter<br>1 Short-chain dehydrogenase/reductase SDR<br>1 Drug resistance transporter (EmrB/QacA)<br>2 Crotonyl-CoA reductase / alcohol dehydrogenase	Decaying wood
	Streptosporangium sp. CA-135522 (GCF_050949235.1)	24	No	1 SARP<br>1 LuxR response regulator	1 Amidohydrolase<br>1 Thymidylate synthase complementing protein<br>1 Methylamine utilization protein MauE<br>1 Redoxin<br>1 ABC transporter<br>1 Signal peptidase (peptidase S26)<br>1 Ferredoxin	Soil under Olea tree

					1 Peptidase C26<br>1 Peptidase_S9<br>1 Enoyl-CoA hydratase/isomerase<br>1 Amino acid permease<br>1 Polyketide cyclase / dehydrase and lipid transport<br>1 Acyl-CoA_dh_N<br>1 Ribosomal RNA adenine dimethylase	
	Streptosporangium sp. CA-250124 (CP191879.1)	30	No	2 GntR<br>1 LysR<br>1 SARP	1 NUDIX domain containing protein<br>1 DUF4440<br>1 HD domain protein<br>1 C-5 cytosine-specific DNA methylase<br>1 *Sac*I restriction endonuclease<br>1 DNA mismatch endonuclease Vsr<br>1 Helix-turn-helix of DDE superfamily endonuclease<br>1 Ornithine/lysine/arginine decarboxylase<br>1 HNH endonuclease<br>1 DUF5997	NS
II	*Solwaraspora* sp. WMMD791 (GCF_029581195.1)	17	No	1 SARP<br>1 TetR<br>1 Helix-turn-helix domain protein	1 FAD linked oxidase domain protein<br>1 Short-chain dehydrogenase/reductase SDR<br>1 ABC transporter<br>1 Amidohydolase<br>1 Inner-membrane translocator<br>1 ABC transporter<br>1 ParA protein	*Ecteinascidia turbinata*
	Lentzea sp. CA-247081 (CP191905.1)	28	NRPS	1 RNA polymerase, sigma-24 subunit (ECF subfamily)<br>1 YjbR<br>1 SARP<br>1 TetR	1 6-O-methylguanine DNA methyltransferase (ribonuclease-like domain)<br>1 Major facilitator transporter<br>1 UDP-glucoronosyl and UDP-glucosyl transferase<br>1 FAD linked oxidase domain protein<br>1 ABC transporter	NS

					1 Acyl-CoA dehydrogenase<br>1 Alpha/beta hydrolase family<br>1 Catalase<br>1 Acetyltransferase (GNAT) domain	
	Umezawaea sp. CA-247087 (CP191890.1)	19	No	1 MarR<br>1 TetR<br>1 LysR<br>1 SARP	1 DUF1348<br>1 SnoaL-like domain protein<br>1 FAD linked oxidase domain protein<br>1 ABC transporter<br>1 DUF4132	NS
	Actinokineospora sp. HUAS TT18 (GCF_051364635.1)	19	NRPS	1 SARP<br>1 MarR<br>1 OsmC-like protein	1 ABC transporter<br>1 FAD linked oxidase domain protein<br>1 Sugar-binding lipoprotein	Soil
	Saccharothrix syringae NRRL B-16468 (GCF_009498035.1)	20	No	2 SARP<br>1 LysR	1 FAD linked oxidase domain protein<br>1 ABC transporter<br>1 Transporter (EamA family)	Soil
	Saccharothrix isguenensis DSM 46885 (GCF_052936305.1)	22	NRPS	1 RNA polymerase, sigma-24 subunit (ECF subfamily)<br>1 LuxR<br>1 SARP	1 DUF1540<br>1 FAD linked oxidase domain protein<br>1 ABC transporter<br>1 DUF4132	Desert soil
**II**	*Saccharothrix espanaensis* DSM 44229 (GCF_000328705.1)	20	No	1 SARP	1 Glycosyl transferase (group 1)<br>1 ABC transporter<br>1 FAD linked oxidase domain protein<br>1 Methyltransferase	Soil
	Actinosynnema sp. CA-254689 (CP191832.1)	18	NRPS	1 SARP	1 Zinc-binding dehydrogenase<br>1 Methyltransferase domain protein<br>1 Acetyltransferase (GNAT) family<br>1 ABC transporter<br>1 FAD linked oxidase domain protein<br>1 O-methyltransferase	NS
	Nonomuraea glycins NBC 01726 (CP109166.1)	18	No	1 Sensor histidine kinase	1 FAD linked oxidase domain protein<br>1 ABC transporter	Soil

				1 LuxR response regulator<br>1 AsrR	2 Crotonyl-CoA reductase / alcohol dehydrogenase	
	Nonomuraea sp. CA-293502 (CP191977.1)	22	NRPS	1 Sensor histidine kinase<br>1 LuxR response regulator<br>1 MarR	1 Acyl-CoA dehydrogenase<br>1 PspC domain protein<br>1 Major facilitator transporter	Rhizospheric soil from Gypsophila
	Kutzneria chonburiensis SMC256 (GCF_028622115.1)	18	NRPS and NAPAA	1 Helix-turn-helix domain protein<br>1 Cold-shock DNA-binding domain protein	1 Alpha/beta hydrolase fold<br>1 Mandelate racemase/muconate lactonizing enzyme<br>1 Acetyltransferase (GNAT) family<br>1 Cytochrome P450<br>1 Glycosyl hydrolase family<br>1 ABC<br>1 Peptidase M1	Soil
	Kutzneria sp. CA-103260 (GCF_018263855.1)	21	NRPS and NAPAA	1 Helix-turn-helix domain<br>1 Cold-shock DNA-binding	2 Cytochrome P450<br>1 Beta-lactamase<br>1 DUF4180<br>1 Alpha/beta hydrolase family<br>1 Mandelate racemase/muconate lactonizing enzyme<br>1 ABC transporter<br>1 Peptidase M1	Soil under *Eucharis bouchei*
	Actinoallomurus sp. CA-150999 (GCF_050942425.1)	21	No	2 LysR<br>1 MerR<br>1 NmrA family protein<br>1 Histidine kinase-like ATPase domain<br>1 Penicillinase repressor	1 NA recombination-mediator protein A<br>1 DUF5753<br>1 Sulfotransferase<br>1 Peptidase family M48<br>1 Short-chain dehydrogenase/reductase SDR	Soil from a waterlogged area in rainforest
